# Enhancing osteogenic differentiation in adipose-derived mesenchymal stem cells with Near Infra-Red and Green Photobiomodulation

**DOI:** 10.1016/j.reth.2023.11.003

**Published:** 2023-11-10

**Authors:** Daniella Da Silva, Anine Crous, Heidi Abrahamse

**Affiliations:** Laser Research Centre, Faculty of Health Sciences, University of Johannesburg, P.O. Box 17011, Doornfontein, Johannesburg, 2028, South Africa

**Keywords:** Adipose-derived mesenchymal stem cells (ADMSC), Osteogenic induction, Photobiomodulation (PBM), Differentiation inducers, Stem cell therapy

## Abstract

Worldwide, osteoporosis is the utmost predominant degenerative bone condition. Stem cell regenerative therapy using adipose-derived mesenchymal stem cells (ADMSCs) is a promising therapeutic route for osteoporosis. Photobiomodulation (PBM) has sparked considerable international appeal due to its’ ability to augment stem cell proliferation and differentiation properties. Furthermore, the differentiation of ADMSCs into osteoblast cells and cellular proliferation effects have been established using a combination of osteogenic differentiation inducers and PBM. This *in vitro* study applied dexamethasone, β-glycerophosphate disodium, and ascorbic acid as differentiation inducers for osteogenic induction differentiation media. In addition, PBM at a near-infrared (NIR) wavelength of 825 nm, a green (G) wavelength of 525 nm, and the novel combination of both these wavelengths using a single fluence of 5 J/cm^2^ had been applied to stimulate proliferation and differentiation effectivity of immortalised ADMSCs into early osteoblasts. Flow cytometry and ELISA were used to identify osteoblast antigens using early and late osteoblast protein markers. Alizarin red Stain was employed to identify calcium-rich deposits by cells within culture. The morphology of the cells was examined, and biochemical assays such as an EdU proliferation assay, MTT proliferation and viability assay, Mitochondrial Membrane Potential assay, and Reactive Oxygen Species assay were performed. The Central Scratch Test determined the cells' motility potential. The investigative outcomes revealed that a combination of PBM treatment and osteogenic differentiation inducers stimulated promising early osteogenic differentiation of immortalised ADMSCs. The NIR-Green PBM combination did appear to offer great potential for immortalised ADMSC differentiation into early osteoblasts amongst selected assays, however, further investigations will be required to establish the effectivity of this novel wavelength combination. This research contributes to the body of knowledge and assists in the establishment of a standard for osteogenic differentiation *in vitro* utilising PBM.

## Highlights

• Immortalised adipose-derived stem cells can develop into various cell types.
• Photobiomodulation combined with differentiation inducers enhances differentiation.
• Green and NIR-G wavelengths stimulated differentiation the most morphologically.
• NIR-G wavelength stimulated the greatest increase in osteogenic marker expression.
• In summary, photobiomodulation application enhances cell differentiation abilities.

## Graphical abstract

The combined use of osteogenic differentiation inducers and photobiomodulation improved the differentiation potential of immortalised adipose-derived mesenchymal stem cells into early osteoblast cells as well as their proliferation potential.

**Figure:**
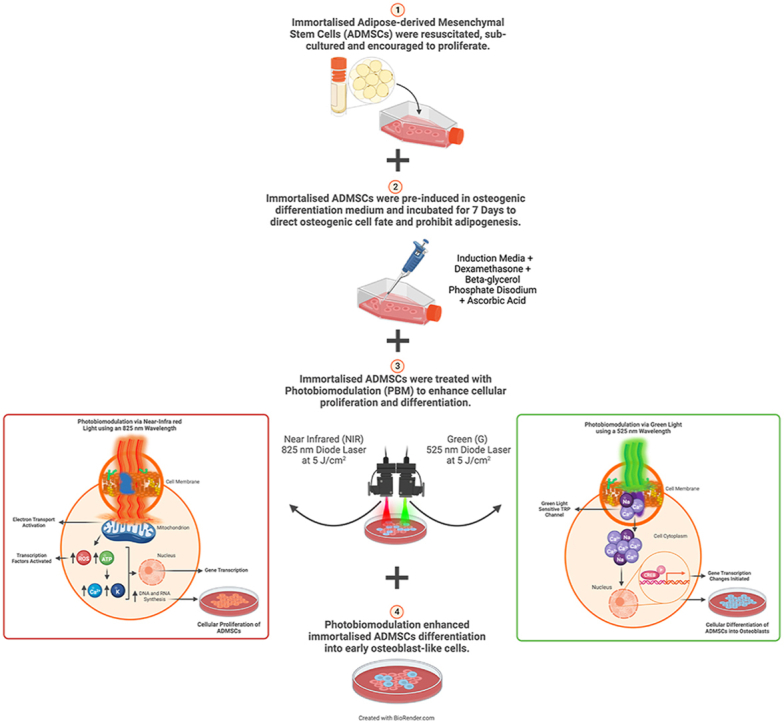

## 1 Introduction

Amid aging populations with extended life expectancies, a global osteoporotic pandemic is on the rise [1]. Osteoporosis is a lifetime skeletal disease defined as a decrease in bone mass, a decrease in bone density, and an overall deterioration of the bone [2]. The development of osteoporosis is linked to a discrepancy between bone resorption and bone development caused by a decrease in bone-forming mature osteoblast populations [3,4]. Presently, osteoporosis treatments intend to prevent osteoporosis disease whilst effectively reducing the possibility of fractures [5]. However, these treatments potentially initiate rare and severe contra-indications when used for extended periods [[6], [7], [8]]. Regenerative medicine (RM) is a promising field of medical science intended to repair functionality and heal tissues or organs that have become diseased, injured, or affected by age-related conditions. At the frontline of RM stands stem cell (SC) therapy, due to the beneficial characteristics of SCs including self-renewal and cell differentiation into various cell types [9].

Adipose-derived mesenchymal stem cells (ADMSCs) are a preferred and ideal cell source for the purposes of RM due to their cell yield abundancy, their proliferation competency, their ability to be morphologically and genetically stable for extended time periods in cell culture, and near-painless isolation from adipose tissues with minimal ethical concerns [[10], [11], [12], [13]]. Notably, previous studies have established immortalised ADMSCs to be an alternate cell type to primary ADMSCs as similar results are produced [14]. Immortalised cell lines offer advantages, such as a lack of ethical controversy, an unlimited supply of material, and are cost effective for experimental purposes. Moreover, immortalised cell lines offer a pure population of cells, which is necessary to deliver a consistent cell sample and reproducible results [15]. It is acknowledged that a steady, immortalised cell line that does not go through aging would be a successful producer of biologically active factors in indefinite quantities compared to primary cell lines, qualifying their use in stem cell therapy to be repeatable and enhanced considering the number of administrations [16]. Adipose-derived mesenchymal stem cells are capable of multi-differentiation into adipocytes, osteoblasts, chondrocytes, and smooth muscle cells [12]. The acceleration of effective osteogenic differentiated ADMSCs *in vitro* to proliferate, differentiate and regulate osteoblast cells requires the presence of multiple biological osteogenic differentiation inducers such as ascorbic acid, insulin-like growth factor-1, bone morphogenetic protein −2 and 1,25 vitamin D3 [17,18]. In combination with biological osteogenic differentiation inducers, chemical differentiation inducers have the capacity to regulate and guarantee the expected fate of mesenchymal stem cell (MSC) differentiation [19]. Dexamethasone, β-glycerol phosphate disodium, calcium phosphate families and hypoxia-inducible factor are frequently added chemical osteogenic differentiation inducers for osteogenic differentiation and assists in the prohibition occurrence of adipogenesis [20]. Adipose-derived mesenchymal stem cells that can effectively differentiate into osteoblast lineages *in vitro*, potentially suggests their ability to migrate, proliferate and differentiate under *in vivo* transplantation circumstances [[21], [22], [23]]. Despite the confirmed studies of biological and/or chemical osteogenic differentiation inducers potential to produce osteogenic differentiation of ADMSCs, it has been observed that ADMSCs favour adipogenic lineages unless strictly controlled [24]. The requirement to control lineage specific differentiation, demands the combinational use of biological and/or chemical osteogenic differentiation inducers and physical stimulation to differentiate ADMSCs into early osteoblasts.

**Fig. 1:**
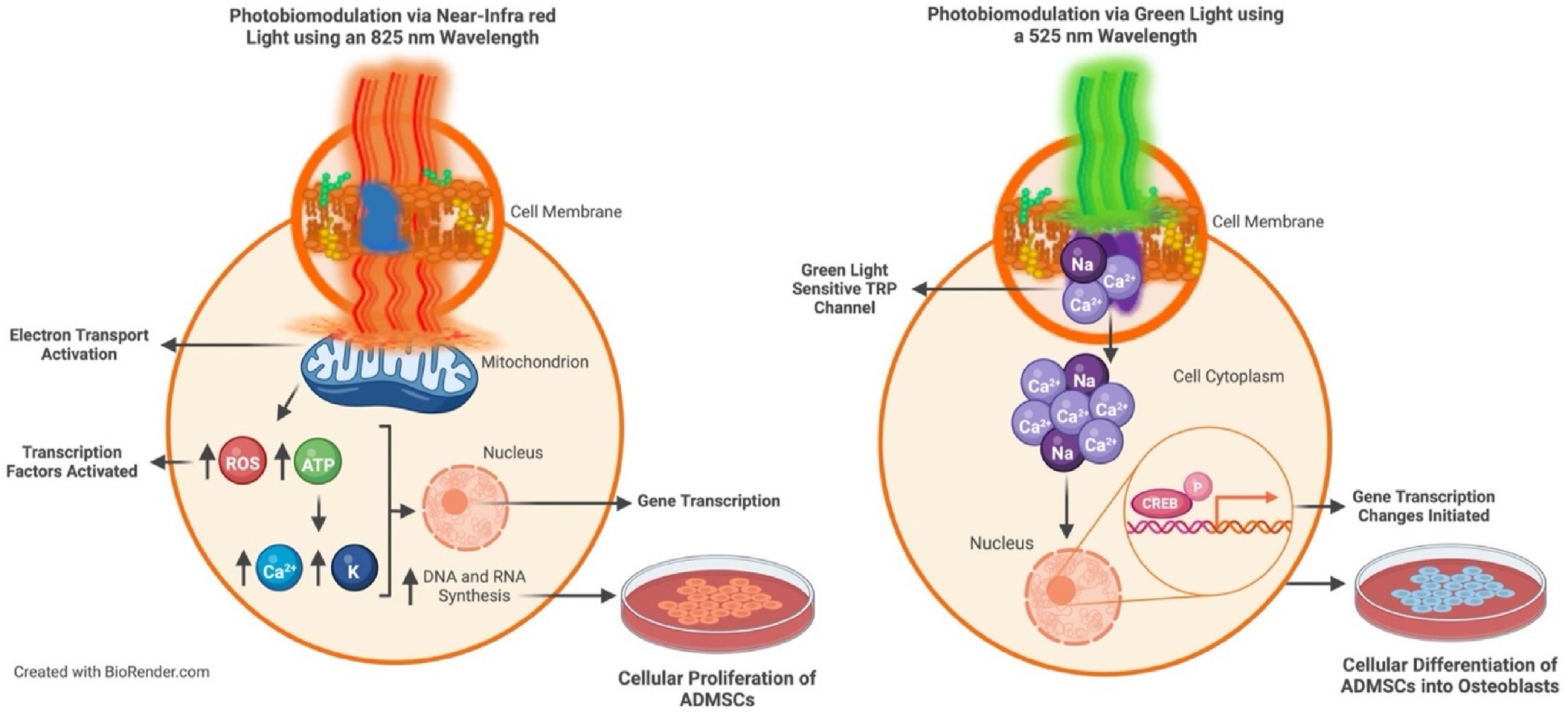
The proposed mechanisms of Near-Infrared and Green light. Photobiomodulation using a Near-Infrared light of 825 nm wavelength penetrates through the cell membrane reaching the mitochondria of the cell. The enzymatic chromophore, CcO, is found within the mitochondria and absorbs the infrared light. The enzyme plays a role in the electron transport chain during ATP production. Increased amounts of ATP bring about an increased gene transcription within the nucleus resulting in increased DNA and RNA synthesis causing the cells to proliferate. Photobiomodulation using a Green light of 525 nm wavelength penetrates the cell membrane and excites the downstream target opsin receptor, the transient receptor potential (TRP) channel. After the application of a stimulus, TRP channels will open and allow an overflow of calcium (Ca2+) ions into the cell cytoplasm. The Ca2+ influx enables calcium/calmodulin dependent kinase II (CAMKII) activity triggering cAMP response element-binding protein (CREB) phosphorylation in the nucleus. The gene transcription changes initiated by CREB are believed to produce the favourable effects of PBM such as cell differentiation.

Photobiomodulation (PBM) is a promising stimulant that uses light energy from coherent or non-coherent light sources in a visible and near-infrared (NIR) range, stimulating endogenous chromophores bringing about photochemical and photophysical reactions [25,26]. Photobiomodulation, either by itself or in combination with biological and/or chemical osteogenic differentiation inducers, speeds up the bone matrix synthesis substantially, elevates osteogenic populations and encourages cell proliferation and differentiation [[27], [28], [29], [30]]. The use of red and NIR light has been suggested to stimulate Cytochrome c Oxidase (CcO) due to the movement of inhibitory nitric oxide resulting in stimulated CcO activity, increasing mitochondrial membrane potential (MMP), and enabling mitochondrial adenosine triphosphate (ATP) production for cell proliferation [31,32]. Recently, the green (G) (495–570 nm) light PBM proposed mechanism is understood to trigger opsin signalling and influence cellular differentiation [33,34]. Due to their excitation by G light, the G-protein coupled receptors, opsins are rapidly becoming significant in the use of phototherapy studies [35] (Fig. 1). Significantly, the combination of different wavelengths on MSC proliferation and differentiation into osteoblasts *in vitro* has rarely been explored [36]. Yet, the outcomes of NIR-G combined wavelengths have not yet been determined.

**Fig. 2:**
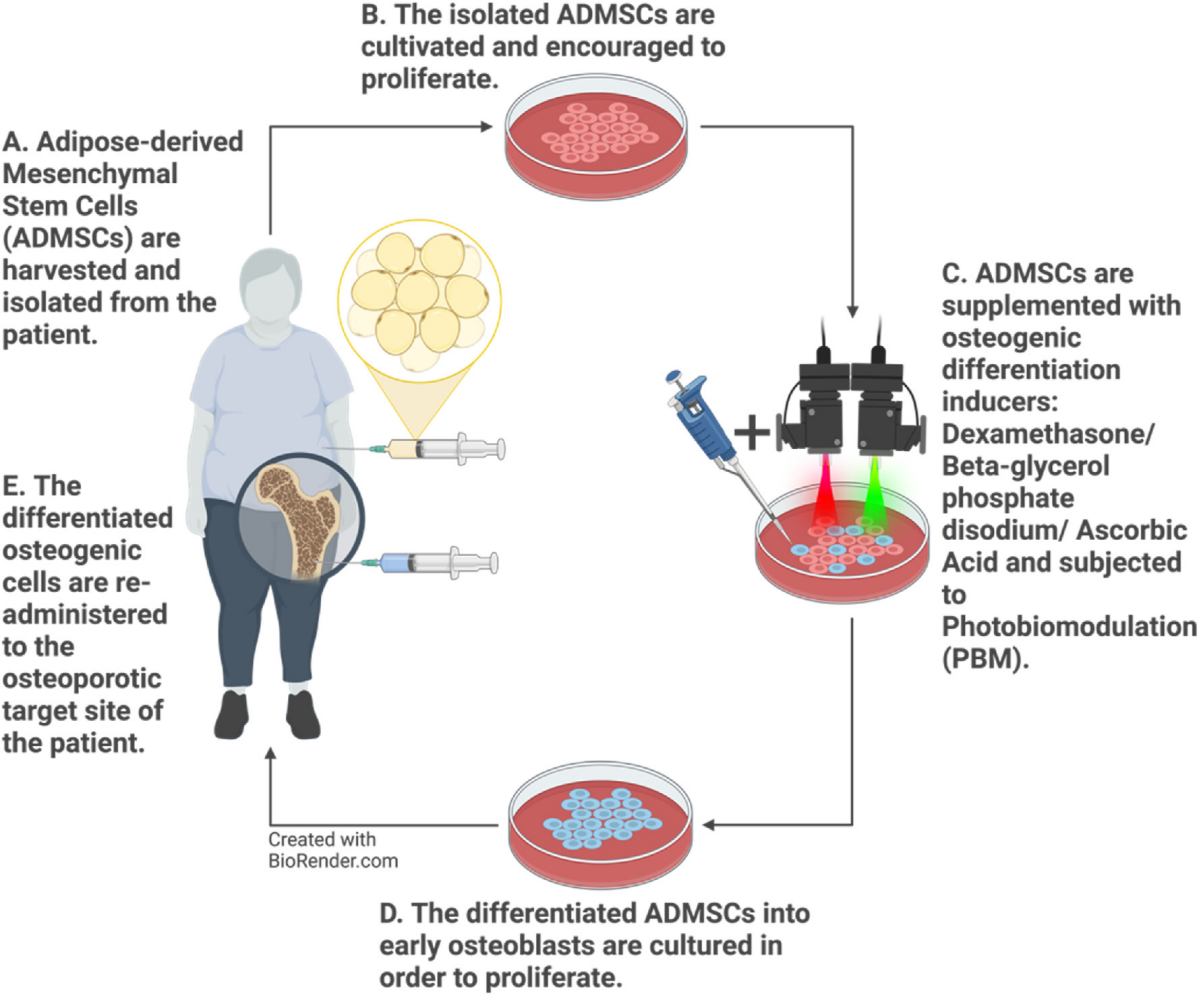
Theoretical clinical application. The isolation of adipose-derived mesenchymal stem cells from the host via a biopsy, from which cells are then stimulated to proliferate and differentiate using various osteogenic differentiation inducers and PBM as a physical stimulant. The post-differentiated osteogenic cells are transplanted back into the host for therapeutic application.

A momentary production of reactive oxygen species (ROS) is anticipated with photobiomodulation application [37] which essentially participates in SC differentiation [38]. However, photoinhibition has been identified when using higher fluences, producing an increased ROS and cell damage [39]. Photoinhibition, often referred to as the biphasic dose response, is a well observed phenomenon [37,40] that states that the positive response eventually reaches its maximum as the light dose (J/cm^2^) is gradually raised. The reaction degrades as the dose is raised more after it reaches its maximum [41,42]. If the light dose is increased even further, a negative response begins to occur, inducing inhibition, which could lead to cellular harm or cell death [43]. A research consensus has indicated that PBM at fluences between 0.1 and 4 J/cm^2^ requires multiple doses and/or exposure intervals to stimulate favourable cellular outcomes [36,[44], [45], [46], [47]]. Currently, limited investigations have reported an increased cell proliferation, viability, and migration at a single 5 J/cm^2^ dose [28,40,47,48] suggesting an optimum influence on cellular function whilst limiting the possibility of photoinhibition. Furthermore, light doses above 7 J/cm^2^ tend to provoke the biphasic dose response subsequently inducing cell death [47,[49], [50], [51]]. However, if the biphasic dose response is avoided, PBM has been shown by several researchers [[52], [53], [54]] to have positive effects on inflammation, oxidative stress, survival, and tissue regeneration [55]. As a result of its virtually non-existent adverse effects, PBM appears to improve treatment outcomes [50].

This *in vitro* study aimed to investigate the effective proliferation and differentiation of immortalised ADMSCs into viable osteoblast lineage cells via the combinational use of osteogenic differentiation inducers and PBM at a NIR wavelength of 825 nm, a G wavelength of 525 nm and NIR-G wavelengths (825 nm and 525 nm) using a single fluence of 5 J/cm^2^. This study intended to add to the standardisation of *in vitro* protocols for the differentiation of ADMSCs into early osteoblasts to result in a safe and sound procedure that can be efficiently translated *in vivo* for the clinical treatment of osteoporosis as a regenerative tool (Escudero et al., 2019) (Fig. 2).

## 2 Material and methods

### 2.1 Cell culture

Immortalised ADMSCs (ASC52telo hTERT, ATCC® SCRC-4000™) were cultured in induction medium containing Dulbecco's Modified Eagle Media (DMEM) (Sigma-Aldrich, South Africa, D5796) supplemented with 10 % foetal bovine serum (FBS Superior) (Biochrom, South Africa, S0615) and 1 % antibiotics: 0.5 % Penicillin-Streptomycin (Sigma-Aldrich, South Africa, P4333) and 0.5 % Amphotericin B solution (Sigma-Aldrich, South Africa, A2942). All cultured cells were maintained in Corning® cell culture flasks (Sigma, South Africa, CLS430639/CLS430641/CLS431080) and incubated at 37 °C in 5 % CO_2_ and 85 % humidity (Heracell™ 150i CO_2_ Incubator, ThermoScientific™, South Africa, 51026280). The cultured immortalised ADMSCs were sub-cultured and maintained in Corning® cell culture flasks in osteogenic induction differentiation media consisting of induction medium supplemented with [50 nM] dexamethasone (Sigma-Aldrich, South Africa, D4902), [10 nM] β-glycerol phosphate disodium (Sigma-Aldrich, South Africa, 50020) and [0.2 mM] ascorbic acid (Sigma-Aldrich, South Africa, A4403) osteogenic differentiation inducers. The cells were incubated for 7 days in osteogenic induction differentiation media prior to irradiation.

### 2.2 Photobiomodulation

Upon confluency, cultured immortalised ADMSCs were seeded at a density of 1 × 10^5^ onto 35 mm diameter culture dishes (Corning®, South Africa, 430165) in 2 mL of osteogenic induction differentiation media and maintained for 24 h to allow adhesion prior to irradiation. After this incubation period, cells were irradiated with a NIR 825 nm Diode Laser (National Laser Centre of South Africa, SN 101080908ADR-1800), G 525 nm Diode Laser (National Laser Centre of South Africa, EN 60825–1:2007) and NIR-G (825 nm and 525 nm) wavelengths all at a single fluence of 5 J/cm^2^. The power of the laser output (mW) was measured using a FieldMate Laser Power Meter (Coherent, South Africa, 1098297). Finally, a High-Sensitivity Thermopile Sensor PM3 (Coherent, South Africa, 1098336) was used to determine the laser irradiation time based on fluency. The laser parameters can be seen in Table 1. The calculation for irradiation time was determined with the formula seen in Equation 1.

$$mW/{cm}^{2}=\frac{mW}{\pi \times \left(r^{2}\right)}$$

$$W/{cm}^{2}=\frac{mW/{cm}^{2}}{1000}$$

$$Time\;\left(s\right)=\frac{J/{cm}^{2}}{W/{cm}^{2}}$$

**Table 1:** Laser irradiation parameters.

Laser	Light Source	Wavelength (nm)	Power Output (mW)	Spot Size (cm^2^)	Power Density (mW/cm^2^)	Intensity (W/cm^2^)	Emission	Fluence (J/cm^2^)	Time of Irradiation (s)
Near-infrared (NIR)	Diode Laser	825	515	9.62	53.53	0.05	Continuous Wave	5	93
Green (G)	Diode Laser	525	553	9.62	57.47	0.06	Continuous Wave	5	87

**Equation 1.** Laser irradiation time. Where *m*W/*cm*^2^ is the power density, *W*/*cm*^2^ is the intensity, and *s* is the exposure time.

### 2.3 Experimental model

**Fig. 3:**
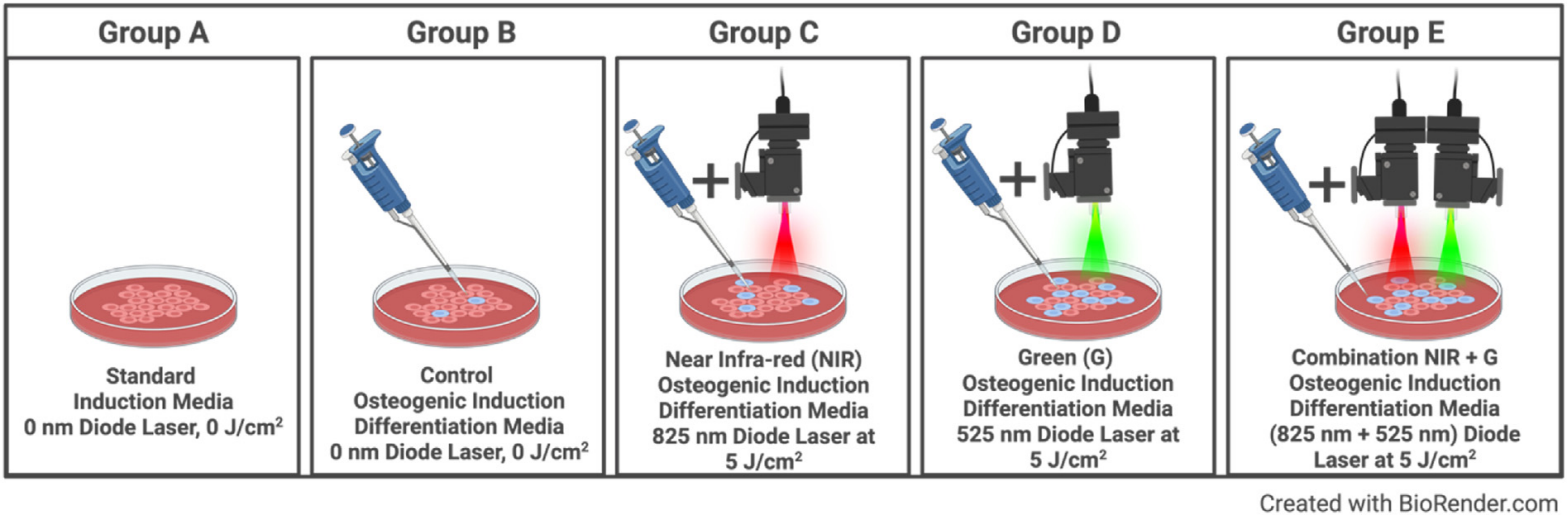
Experimental cell culture model. Cells in Group A received neither osteogenic differentiation inducers nor photobiomodulation treatment. Cells in Group B received osteogenic differentiation inducers but did not receive photobiomodulation treatment. Cells in Group C received both osteogenic differentiation inducers and Near Infra-red photobiomodulation treatment at an 825 nm wavelength at a 5 J/cm^2^ dose. Cells in Group D received both osteogenic differentiation inducers and Green photobiomodulation treatment at a 525 nm wavelength at a 5 J/cm^2^ dose. Cells in Group E received both osteogenic differentiation inducers and Near Infra-Red-Green combined photobiomodulation treatment at 825 nm and 525 nm wavelengths at a 5 J/cm^2^ dose.

This experiment was divided into 5 groups: Group A are cells that did not receive osteogenic differentiation inducers nor PBM treatment, Group B are cells that received osteogenic differentiation inducers within cell culture but did not receive PBM treatment, Group C are cells that received osteogenic differentiation inducers within cell culture and PBM treatment at a 825 nm wavelength at 5 J/cm^2^, Group D are cells that received osteogenic differentiation inducers within cell culture and PBM treatment at a 525 nm wavelength at 5 J/cm^2^, and Group E are cells that received osteogenic differentiation inducers within cell culture and PBM treatment at combined wavelengths of 825 nm and 525 nm at 5 J/cm^2^. The experimental cell culture model has been represented in Fig. 3.

**Fig. 4:**
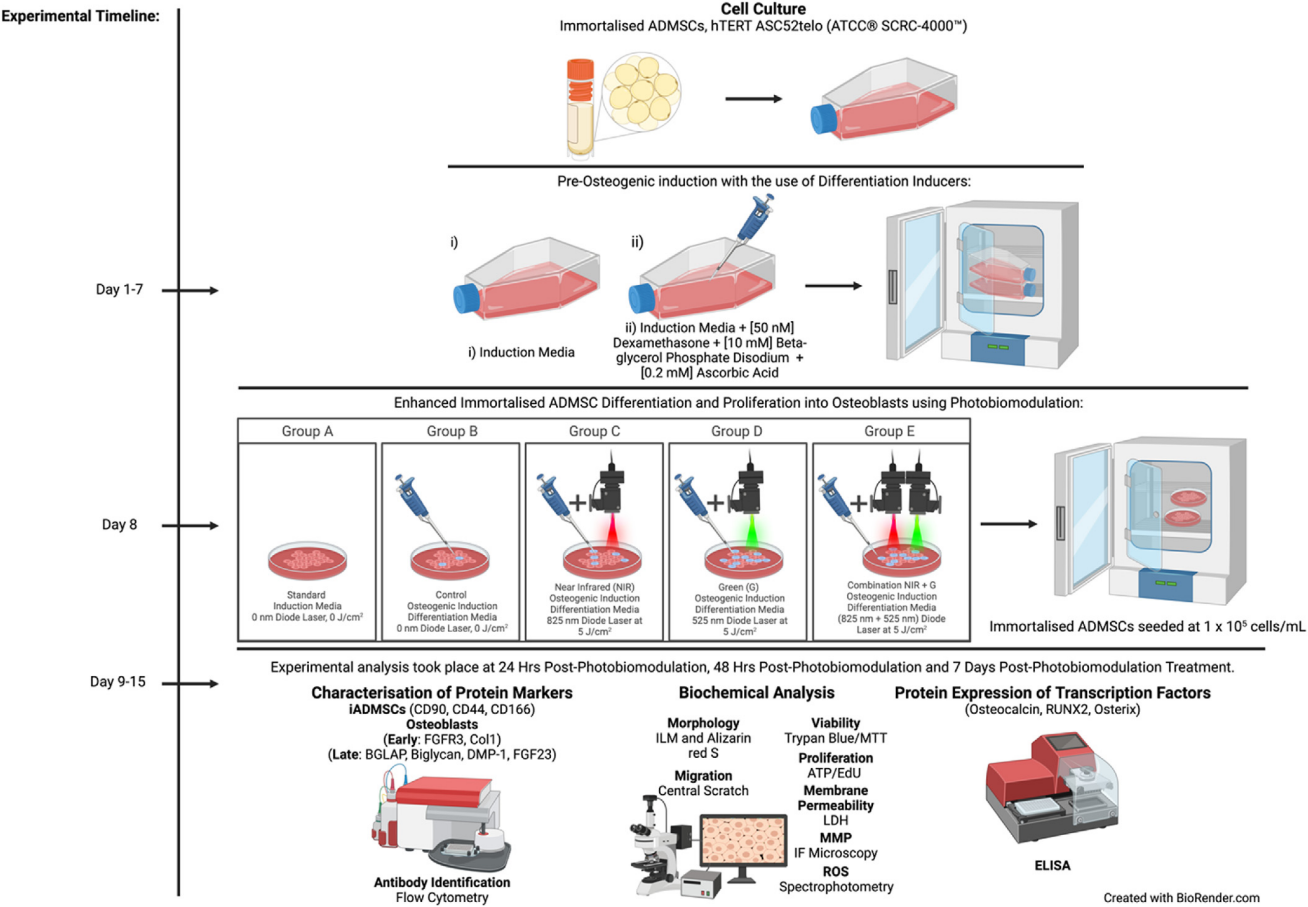
Experimental methodology. Immortalised adipose-derived mesenchymal stem cells were resuscitated and sub-cultured until desirable confluency was attained. Dexamethasone, β-glycerol phosphate disodium, and ascorbic acid differentiation inducers were used to initiate and direct osteogenic differentiation during a several days incubation. The cells were irradiated with a single fluence of 5 J/cm^2^ at wavelengths of Near-Infrared 825 nm, Green 525 nm, and their combination wavelengths for enhanced cellular differentiation into osteoblasts and cellular proliferation. An experimental standard in which cells received neither osteogenic differentiation inducers nor photobiomodulation treatment, as well as an experimental control in which cells received only osteogenic differentiation inducers and not photobiomodulation treatment, were included. The cell samples were collected at 24 h, 48 h and 7 days post-irradiation. Cell characterisation, via the use of flow cytometry as a quantitative assay, identified early protein markers (FGFR-3 and Col-1) and late osteogenic protein markers (Osteocalcin, Biglycan, DMP-1 and FGF-23). Calcium deposit identification was recognised by Alizarin red Stain morphology. Morphological analysis was seen by inverted light microscopy. Cell migration was investigated via the ‘Central Scratch Test’ method. Biochemical analysis such as cell viability, cell proliferation, membrane permeability, mitochondrial membrane potential and reactive oxygen species were determined. The protein expression of osteogenic early (RUNX2) and late (Osteocalcin and Osterix) transcription factors were identified using ELISA.

### 2.4 Osteogenic differentiation

Osteoblast differentiation was characterised using early osteogenic protein surface markers fibroblast growth factor receptor-3 (FGFR-3) (Sigma-Aldrich, South Africa, SAB4500888) and collagen 1 (Col-1) (Sigma-Aldrich, South Africa, SAB1402151) and late osteogenic protein surface markers osteocalcin (BGLAP) (Sigma-Aldrich, South Africa, WH0000632M1), biglycan (BGN) (Sigma-Aldrich, South Africa, WH0000633M1), dentin matrix protein-1 (DMP-1) (Sigma-Aldrich, South Africa, SAB1402752) and fibroblast growth factor-23 (FGF-23) (Sigma-Aldrich, South Africa, SAB1406641).

#### 2.4.1 Flow cytometry

The differentiated immortalised ADMSCs were fluorescently labelled using the secondary antibody-conjugation technique at 24 h and 7 days post-PBM treatment. Primary rabbit anti-human antibody anti-FGFR-3 and primary mouse anti-human antibodies anti-Col-1, anti-BGLAP, anti-BGN, anti-DMP-1 and anti-FGF-23 were used. The treated immortalised ADMSCs were placed in suspension, washed with 1 mL PBS (100 mL 10X PBS (Sigma-Aldrich, South Africa, P5493)/900 mL Milli-Q water) using centrifugation in a Heraeus Fresco™ 17 Microcentrifuge (ThermoFisher Scientific™, South Africa, 75002402) at 4 °C at 4000×*g* for 5 min and resuspended in 1 mL ice-cold washing buffer (PBS/Bovine Serum Albumin (BSA)/Sodium azide buffer). The cells were incubated with 100 μL of 4 % formaldehyde (Sigma-Aldrich, South Africa, 47608-250 ML-F) for 15 min at room temperature and followed by gentle vortexing. The cells were incubated with 100 μL primary rabbit anti-human antibodies and a primary mouse anti-human antibodies in 1 mL working buffer (2 % BSA in PBS/BSA/Sodium azide buffer) for 30 min at 4 °C. These cells were washed once with 1 mL washing buffer, centrifuged as above, and incubated with 100 μL secondary fluorescently labelled antibodies, FITC Goat anti-rabbit (Santa Cruz Biotechnology, South Africa, sc-2012) and FITC Goat anti-mouse (NovusBio, South Africa, NB720–F) in 1 mL working buffer for 30 min at 4 °C in the dark. The cells were washed twice with 1 mL washing buffer, centrifuged as above, and resuspended in 500 μL PBS. Antigenic detection was identified using the BD Accuri™ C6 Flow Cytometer (BD 468 Biosciences, USA, BD ACCURI C6) to detect the fluorescent probe on the conjugated antibody, indicating if the cells are antibody positive or negative.

#### 2.4.2 Enzyme-linked immunosorbent assay

The early (RUNX2) and late (Osterix and Osteocalcin) osteogenic markers were identified and quantified using the Human Core Binding Factor alpha1 CBFA1/RUNX2 (Biocom Africa, South Africa, CSB-E12935h), the Osterix (OSX) (Biocom Africa, South Africa, SEN863Hu) and Human Osteocalcin (ThermoFisher Scientific™, South Africa, KAQ1381) ELISA kits, respectively. Each sample included three repeats with technical duplicates of each sample. The assays use monoclonal antibodies directed against distinct epitopes of the RUNX2, Osterix and Osteocalcin proteins. A 100 μL standards and samples react with the capture monoclonal antibody coated on the microplate well and with a monoclonal antibody labelled with horseradish peroxidase (HRP). After 1 h of incubation to allow the formation of an antigen-antibody sandwich, the microplate was washed with 350 μL of wash solution to remove unbound enzyme labelled antibodies. Bound enzyme labelled antibodies were measured via a chromogenic reaction where 100 μL chromogenic solution was added and incubated. The reaction was stopped with 50 μL of stop solution and the microplate was read at an absorbance of 450 nm using the VICTOR Nivo™ plate reader (PerkinElmer, USA, HH3522019094).

#### 2.4.3 Alizarin red stain

An indicator of ADMSC differentiation into osteoblast lineage cells is the deposition of calcium located within the extracellular matrix. In the presence of calcium, an alizarin red S-calcium complex forms in a chelation process resulting in the visibly, bright red stain identifying calcium deposits within the cellular matrix. The immortalised ADMSCs were cultured at a seeding concentration of 1 × 10^5^ cells/mL in 2 mL complete osteogenic induction differentiation medium on heat-sterilised glass coverslips laid on 35 mm diameter non-treated culture dishes (Corning®, South Africa, CLS430588). Cells were incubated at 37 °C and allowed to attach to the coverslip for 24 h. The cells were washed thrice with 1 mL PBS by pipetting and fixed in 500 μL 4 % formaldehyde for 15 min at room temperature. The cells were then washed thrice with 1 mL deionized H_2_O by pipetting and stained with 1 mL 40 mM Alizarin red S (Sigma-Aldrich, South Africa, A5533) solution for 25 min at room temperature. After staining, the cells were rinsed five times with 1 mL PBS by pipetting to remove any excess stain solution and were viewed using inverted light microscopy (Olympus, South Africa, CKX41) and captured with a microscope-connected digital camera (Olympus, South Africa, SC30) that uses the Olympus CellSens Imaging Software program where bright red to orange dots indicate the presence of calcification deposits.

### 2.5 Cell morphology

#### 2.5.1 Inverted light microscopy

The distinctions in morphology were recorded and analysed 24 h, 48 h and 7 days after irradiation via inverted light microscopy (Olympus, South Africa, CKX41) and captured with a microscope-connected digital camera (Olympus, South Africa, SC30) that uses the Olympus CellSens Imaging Software program.

### 2.6 Cell proliferation and viability

#### 2.6.1 Cell motility: “central scratch test” method

Cell motility was analysed using the ‘central scratch’ method. Cells were cultured in 35 mm diameter treated culture dishes and incubated overnight at 37 °C and 5 % CO_2_. A central scratch, with the aid of a pre-set grid and focal planes, was made prior to irradiation using a sterile P-200 pipette tip, the cells were then washed with 1 mL Hank's balanced salt solution (HBSS) and 2 mL of osteogenic induction differentiation medium was added. The cell motility was observed, using set positions on the focal plane, by an Inverted Microscope (Olympus, South Africa, CKX41) and captured using a digital camera (Olympus, South Africa, SC30). Images were taken at 24 h, 48 h and 7 days post-irradiation.

#### 2.6.2 EdU cell proliferation and cycle analysis

Cellular proliferation monitors the health of a cell and cycle analysis through DNA synthesis evaluation by EdU (5-ethynyl-2′-deoxyuridine) (Sigma-Aldrich, South Africa, BCK-FC488). The cells are suspended in their osteogenic induction differentiation medium where 10 μM of EdU was added to the samples and incubated at 37 °C and 5 % CO_2_ for 2 h. The incubation medium was removed, and the cells were washed with 3 mL of 1 % BSA (Sigma-Aldrich, South Africa, A2153) in PBS. The cells were then sedimented at 4 °C at 4000×*g* for 5 min. Followed by 100 μL of the fixative solution added to the cells and incubated at room temperature for 15 min in the dark. The fixative solution was removed, the cells were then washed with 3 mL of 1 % BSA in PBS and centrifuged. The cell pellet was dislodged and resuspended in 100 μL of 1x saponin-based permeabilisation buffer in PBS. The EdU assay (Sigma-Aldrich, South Africa, BCK-FC488) cocktail was prepared and incubated for 30 min at room temperature in the dark. The cells were washed with 3 mL of 1x saponin based permeabilisation and wash reagent and centrifuged. The pellet was dislodged again and 500 μL of 1x saponin-based permeabilisation and wash reagent was added to the cells. The cells were detected using the BD Accuri™ C6 Flow Cytometer (BD 468 Biosciences, USA, BD ACCURI C6) at a low flow rate, excitation of 497 nm, emission of 516 nm and a green filter.

#### 2.6.3 MTT cell proliferation and viability

The MTT assay (Sigma-Aldrich, South Africa, TOX1) is an indicator of cellular viability, proliferation, and cytotoxicity via cellular metabolic activity measurement. This colourimetric assay occurs when metabolically active cells reduce the yellow tetrazolium salt (3-(4,5-dimethylthiazol-2-yl)-2,5-diphenyltetrazolium bromide) or MTT to purple formazan crystals which when dissolved, results in the quantifiable coloured solution. The cells were seeded at 1 × 10^5^ cells/mL onto 35 mm diameter treated culture dishes (Corning®, South Africa, CLS430165) where reconstituted MTT labelling reagent (Sigma-Aldrich, South Africa, M − 5655) at a volume equal to 10 % of the culture medium volume was added before being incubated for 3 h in a humidified atmosphere of 5 % CO_2_ at 37 °C. A volume of MTT solubilisation solution (Sigma-Aldrich, South Africa, M − 8910) equal to the original culture medium volume was added into each well and gently mixed at 25 rpm on a wave motion mixer (Heidolph, Germany, Polymax 1040) for 2 min to improve dissolution of the MTT formazan crystals. The sample was then measured in a flat-bottomed Corning® 96 Well Clear Polystyrene Microplate (Sigma, South Africa, CLS3370) at a recommended absorbance reading of 570 nm using the VICTOR Nivo™ (PerkinElmer, USA, HH3522019094).

### 2.7 Cell cytotoxicity

#### 2.7.1 Mitochondrial membrane potential

Mito Red (Sigma-Aldrich, South Africa, 53271) dye is rhodamine-based that localizes and interacts with the mitochondria revealing the mitochondrial membrane potential via red fluorescent emission. Cells were seeded at a density of 1 × 10^5^ cells/mL on heat-sterilised glass coverslips laid on 35 mm diameter non-treated culture dishes (Corning®, South Africa, CLS430588) and incubated overnight at 37 °C and 5 % CO_2_. The cells were washed with 1 mL HBSS (Sigma-Aldrich, South Africa, 55021C) and incubated with 1 mL [50 nM] Mito Red working solution for 45 min at 37 °C. The cells were washed with 1 mL PBS and fixed with 1 mL 4 % formaldehyde for 15 min at room temperature. Following fixation, the cells were rinsed with 1 mL PBS and mounted onto clean glass slides using Flouromount™ aqueous medium. Images of the fluorescent in-tensity of the mitochondrial membrane potential were obtained using a fluorescence microscope (Olympus, South Africa, BX41) with a rhodamine filter and the Olympus CellSens Imaging Software program.

#### 2.7.2 Reactive oxygen species detection

Reactive oxygen species are generated because of the reduction of oxygen during aerobic respiration and by various enzymatic systems within the cell. At physiological levels, ROS contribute to cell signalling and host defence. Increased ROS generation, above the detoxification capacity of the biological system, results in oxidative stress and cellular damage. The Fluorometric Intracellular ROS Kit (Sigma-Aldrich, South Africa, MAK142) was used to determine the production of ROS. Cells were seeded at a density of 1 × 10^6^ cells/mL into clean 5 mL polystyrene round-bottom tubes (Falcon, South Africa, 352052). To the cells were then added 500 μL ROS Detection Reagent Stock Solution and incubated for 30 min in a 5 % CO_2_, 37 °C incubator. The fluorescent intensity was determined with a BD Accuri™ C6 Flow Cytometer (BD 468 Biosciences, USA, BD ACCURI C6).

The methodology of this *in vitro* investigation has been illustrated in Fig. 4.

### 2.8 Statistical analysis

For statistical analysis, biochemical assays included triplicate biological repeats with technical duplicates. Spectrometry experiments were conducted using a blank sample deducted from the related data collected. The statistical analysis was performed using SigmaPlot version 12 and version 14. Error bars represent the median (SEM) (n = 3). The data were evaluated by a student t-test and a one-way ANOVA. The statistical variances amongst the experimental groups were denoted on the figures as P < 0.05 (∗), P < 0.01 (∗∗) and P < 0.001 (∗∗∗). When comparing experimental groups to the standard, the significance was denoted with a black star (**∗)**, when comparing PBM groups to the control the significance was denoted with a red star (**∗**) and when comparing experimental PBM groups amongst each other, significances were denoted with a turquoise star (**∗**). Data collected from alizarin red stain and motility morphology were quantitatively analysed using Image J, a public Java domain image processing program (National Institute of Health Bethesda, MD USA).

## 3 Results and discussion

### 3.1 Osteogenic differentiation

#### 3.1.1 Flow cytometry

**Fig. 5:**
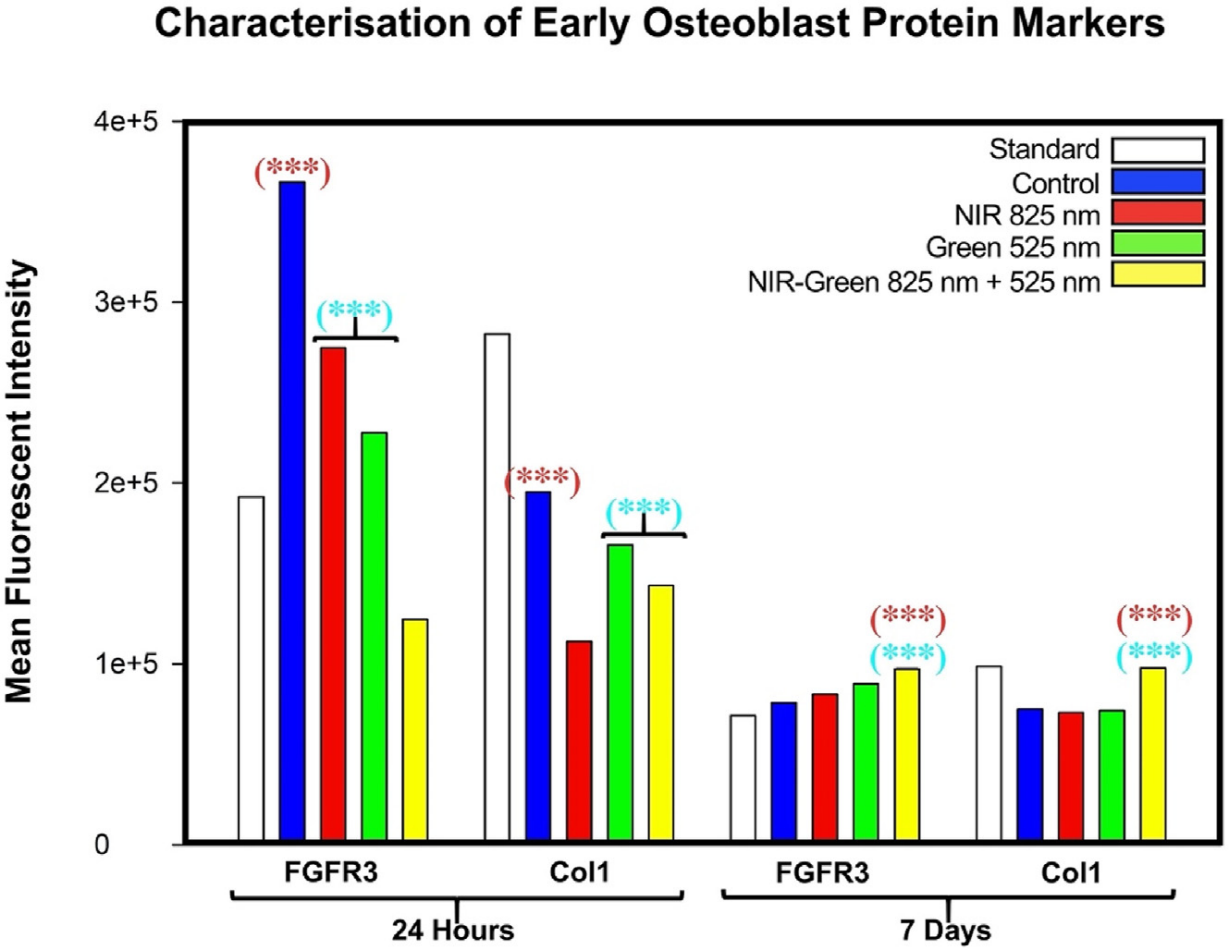
Characterisation of early osteogenic surface markers, FGFR-3, and Col-1, investigated at 24 h and 7 days post-photobiomodulation irradiation. A statistically significant increase at 24 h post-photobiomodulation treatment in FGFR-3 marker expression by the control, Near-Infrared and Green photobiomodulation groups compared to the Near Infrared-Green experimental photobiomodulation group occurred. A statistically significant increase in Col-1 marker expression by the control, Green and Near Infrared-Green photobiomodulation was identified at 24 h post-irradiation. At 7 days post-photobiomodulation treatment, a statistically significant increase in FGFR-3 and Col-1 marker expression by Near Infrared-Green photobiomodulation compared to the control group and amongst the experimental photobiomodulation groups was observed.

**Fig. 6:**
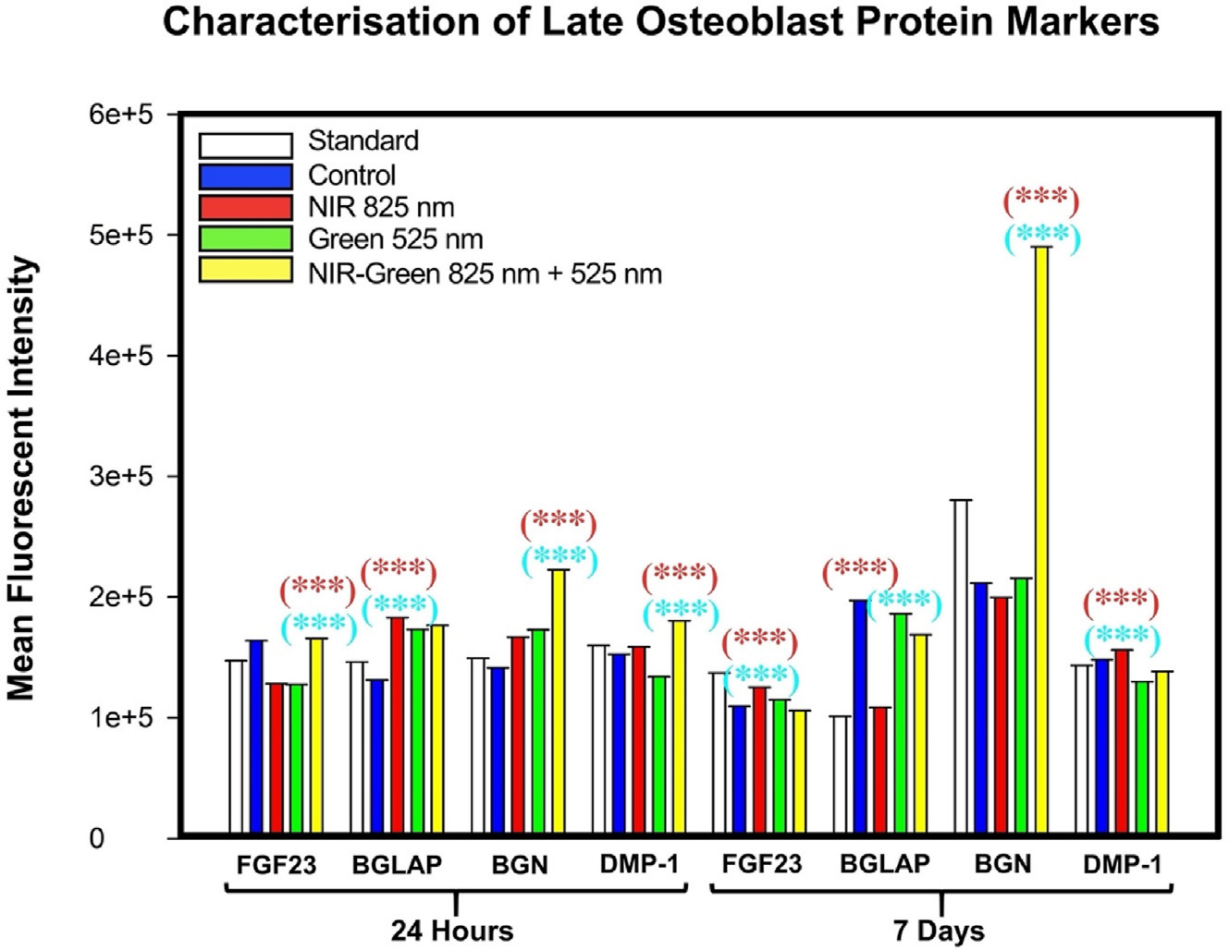
Characterisation of late osteogenic surface markers, FGF-23, BGLAP, BGN and DMP-1, investigated at 24 h and 7 days post-photobiomodulation irradiation. A statistically significant increase at 24 h post-photobiomodulation treatment in FGF-23, BGN and DMP-1 marker expression by Near Infrared-Green photobiomodulation compared to the control group and amongst the experimental photobiomodulation groups was identified. A statistically significant increase in BGLAP marker expression by Near Infra-red photobiomodulation was identified at 24 h post-irradiation compared to the control and the other experimental photobiomodulation groups occurred. At 7 days post-photobiomodulation treatment, a statistically significant increase in FGFR-3 and DMP-1 marker expression by Near Infra-red photobiomodulation compared to the control group and amongst the experimental photobiomodulation groups was observed. A statistically significant increase in BGLAP marker expression by the control group and Green photobiomodulation at 7 days post-irradiation treatment was identified. A statistically significant increase was observed in BGN marker expression at 7 days post-photobiomodulation by the Near Infrared-Green photobiomodulation group compared to the control and other photobiomodulation treatment groups.

Flow cytometry characterisation was used to detect the expression of osteogenic surface markers, where the mean fluorescent intensity is represented as a bar graph. Thus, flow cytometric analysis in Fig. 5 indicated that early surface osteogenic markers, FGFR-3 and Col-1 demonstrated a statistically significant increase at 24 h post-PBM treatment by the control, NIR and G PBM and the control, G and NIR-G PBM, respectively. At 7 days post-PBM treatment, a statistically significant increase was identified in FGFR-3 and Col-1 marker expression by NIR-G PBM compared to the control group and amongst the experimental PBM groups. Fekrazad et al. (2018) had identified Col-1 stimulation when treated with Red PBM, Blue PBM and G PBM at a fluence of 4 J/cm^2^ at 12 days post-PBM treatment [36] while Bölükbaşı Ateş et al. (2017) identified a down regulation of Col-1 at 7 days post-PBM at a wavelength of 809 nm and fluencies of 0.5 and 1 J/cm^2^ [45]. Further flow cytometry investigation determined the presence of late osteogenic surface markers (Fig. 6). Analysis identified a statistically significant increase at 24 h post-PBM treatment in FGF-23, BGN and DMP-1 marker expression by NIR-G PBM compared to the control group and amongst experimental irradiation groups. A statistically significant increase in BGLAP marker expression by NIR PBM was identified at 24 h post-irradiation compared to the control and the other experimental photobiomodulation groups occurred. At 7 days post-PBM treatment, a statistically significant increase in FGFR-3 and DMP-1 marker expression was observed by NIR PBM compared to the control group and amongst the experimental groups. A statistically significant increase in BGLAP marker expression by the control group and G PBM at 7 days post-irradiation treatment was identified. A statistically significant increase was identified in BGN marker expression at 7 days post-photobiomodulation by the NIR-G PBM group compared to the control and the other PBM treatment groups NIR-G PBM to have increased DMP-1 and BGN protein expression and G PBM increased BGLAP and FGF-23 protein expression at 7 days post-PBM treatment. The increased protein expression identified as early as 7 days post-PBM treatment is suggestive of immortalised ADMSC differentiation into early late-stage osteoblast cell development. However, previous studies expressed a decrease in late osteogenic protein expression, particularly osteocalcin, when exposed to G PBM and NIR PBM respectively [36,56]. Another study identified an upregulation of osterix, a late osteogenic marker, in bone marrow stromal cells at a wavelength of 808 nm and continuous fluence of 64 J/cm^2^ [57].

#### 3.1.2 Enzyme-linked immunosorbent assay

**Fig. 7:**
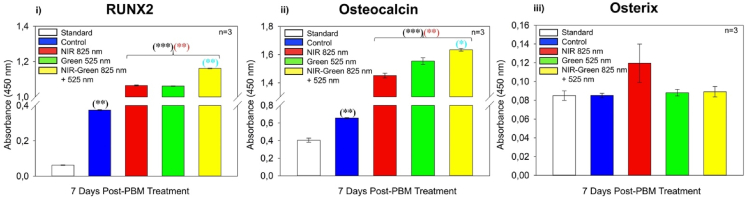
Protein expression of i) RUNX2, ii) Osteocalcin and iii) Osterix using ELISA analysis. A statistically significant increase at 7 days post-photobiomodulation treatment in RUNX2 and Osteocalcin protein expression by Near Infrared-Green photobiomodulation compared to the control group and amongst the experimental photobiomodulation groups.

The protein expression of osteogenic transcription factors, RUNX2, Osteocalcin and Osterix, were identified via ELISA (Fig. 7). A statistically significant increase compared to the standard (P < 0.001) and compared to the control (P < 0.01) in RUNX2 (Fig. 7 i)) was identified. The NIR-G PBM group indicated a statistically significant increase (P < 0.01) in RUNX2 protein expression compared to NIR PBM and G PBM experimental groups. Osteocalcin protein expression (Fig. 7 ii)) identified a statistically significant increase in all experimental groups compared to the standard (P < 0.001) and compared to the control (P < 0.01). The NIR-G PBM demonstrated a statistically significant increase (P < 0.05) in the expression of osteocalcin compared to other PBM groups. Osterix protein was not expressed in this investigation (Fig. 7 iii)). Similar studies differed in comparison to this investigation for genetic expression, as an upregulation had been identified in osteocalcin by Red PBM and G PBM wavelengths at 14 days and 21 days post-PBM treatment [33,36]. Another study identified an increased RUNX2 expression and Osterix expression when exposed to a 540 nm G PBM at 21 days compared to 660 nm red light PBM at 3 J/cm^2^ [33]. Notably, other studies have not investigated the effects of NIR-G PBM combination wavelengths for cellular differentiation making this finding novel. There were no significant findings identified in Osterix, an osteocyte marker, suggesting that these differentiated cells have begun early osteogenic differentiation and have not yet matured.

#### 3.1.3 Alizarin red stain

**Fig. 8:**
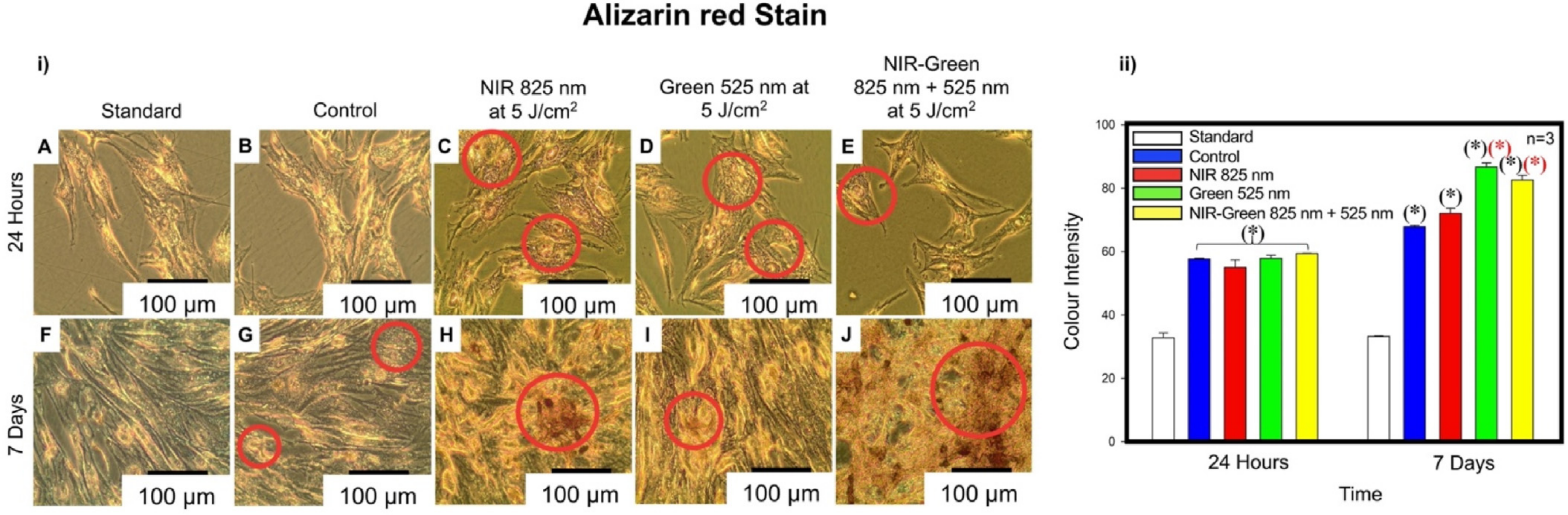
i) Morphological characterisation of cells using an Alizarin red Stain. ii) Quantification of colour intensity of Alizarin red Stain. i) The identification of bright orange deposits at 24 h and 7 days post-photobiomodulation treatment indicative of calcium deposition presence, which is suggestive of osteogenic differentiation (C, D, E, H, I and J). ii) A statistically significant increase occurred at 7 days post-photobiomodulation treatment in Alizarin red Stain colour intensity by Green photobiomodulation and Near Infrared-Green photobiomodulation compared to the control group.

An Alizarin red Stain identifies calcium depositions within the extracellular matrix of the cell. Calcium deposits appear a bright, orange colour in morphological analysis. This investigation identified cells cultured in osteogenic induction differentiation media and stained with Alizarin red demonstrated visibly, bright orange calcium nodules amongst the control and all experimental PBM groups at 24 h and 7 days post-PBM treatment (Fig. 8 i)). This is suggestive of immortalised ADMSC differentiation into osteoblasts as the osteogenic induction differentiation media has encouraged differentiation and PBM has enhanced the cell differentiation activity due to the greater presence of calcium deposits visible. The Bölükbaşı Ateş et al. (2020) study had identified a visibly significant presence of calcium nodules with an Alizarin red Stain on their 14th day of experimental analysis at a wavelength of 809 nm at fluencies of 0.5, 1 and 2 J/cm^2^ [56]. Additional differentiation studies identified a higher amount of calcium deposits in differentiated MSCs irradiated at 2.5 J/cm^2^ using a Gy laser 21 days post-irradiation [58], a significant increase in calcification of cells irradiated with an Er: YAG laser at a wavelength of 2.94 μm at 3.3 J/cm^2^ at 7 days post-irradiation [59] and at 21 days post-irradiation at a wavelength of 650 nm an increase in calcium deposits was identified in the differentiation of human periodontal ligament SCs [60]. The colour intensity of the bright orange calcium nodules was quantified using Image J. A statistically significant increase compared to the standard (P < 0.05) at 24 h and 7 days and compared to the control (P < 0.05) at 7 days amongst the G PBM and NIR-G PBM experimental groups (Fig. 8 ii)) was identified.

### 3.2 Cell morphology

#### 3.2.1 Inverted light microscopy

**Fig. 9:**
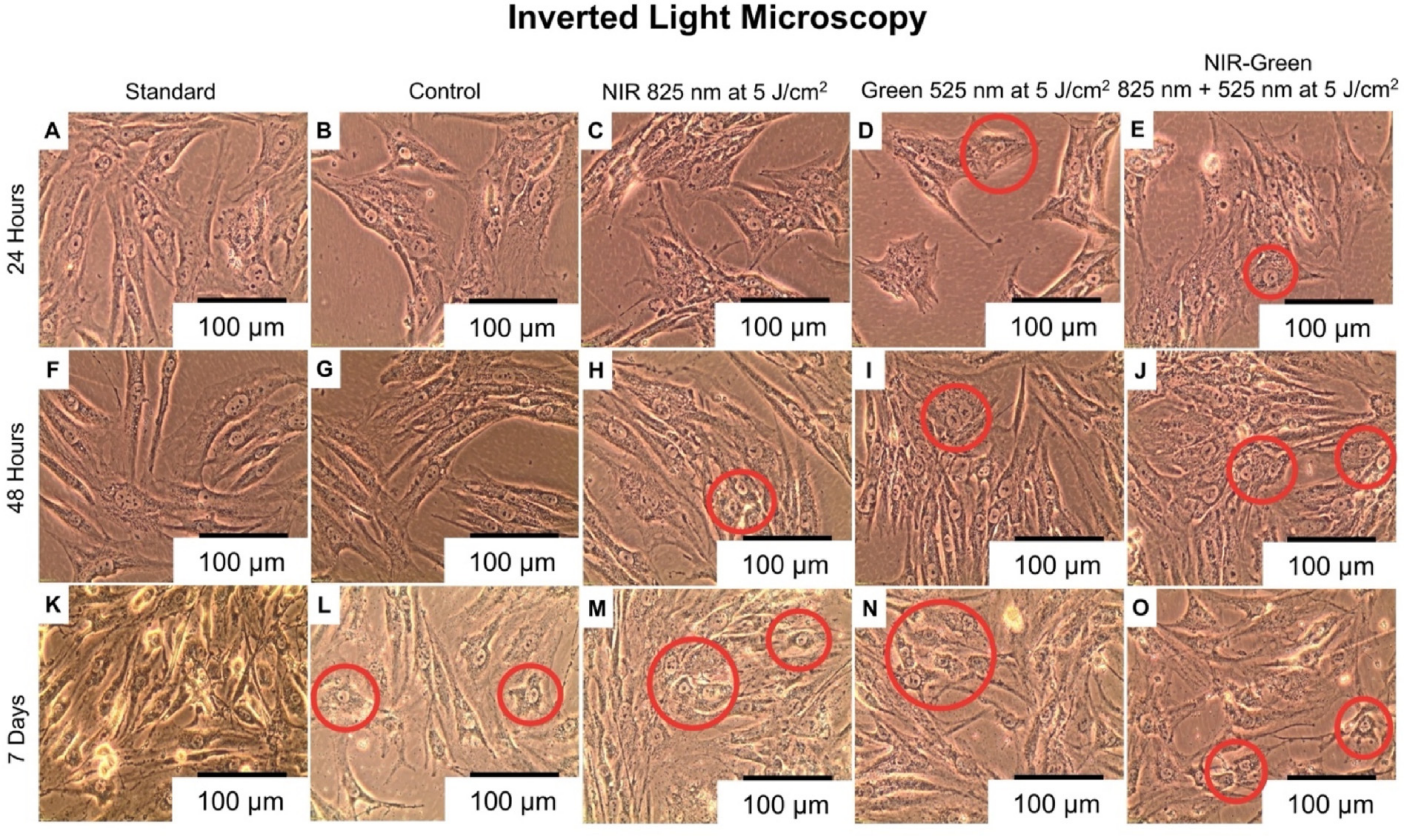
Morphology of immortalised adipose-derived mesenchymal stem cell differentiation 24 h, 48 h, and 7 days post-photobiomodulation treatment using inverted light microscopy. The cell morphology had become rounded and/or shorter spindle shaped at 24 h post-photobiomodulation treatment (D and E), at 48 h post-photobiomodulation treatment (H, I and J) and at 7 days post-photobiomodulation treatment (L, M, N and O). The noticeable changes in cell morphology are similar in appearance to the characteristic cell shape of osteoblast-like cells, suggestive of cell differentiation.

Immortalised ADMSCs are characteristically thin, and spindle shaped. Often, ADMSCs are observed to have a similar morphology to fibroblast cells [61]. A noticeable change in cell morphology (Fig. 9) occurred amongst the G and NIR-G-treated cell groups at 24 h, amongst the NIR, G and NIR-G treated cell groups at 48 h and amongst the control, NIR, G and NIR-G treated cell groups at 7 days post-PBM treatment. The cell shape had become rounded and/or a shorter spindle shaped cell morphology, similar in appearance to the characteristic osteoblast-like cell shape, as identified by previous research [62] and a loss in the visibly thin and longitudinal initial ADMSC cell shape. Another study identified changes in ADMSC morphology at 7 days in the presence of osteogenic differentiation inducers [61]. The noticeable changes in cell morphology suggests the cell differentiation of immortalised ADMSCs into early osteoblasts.

### 3.3 Cell proliferation and viability

#### 3.3.1 Cell motility: “central scratch test” method

**Fig. 10:**
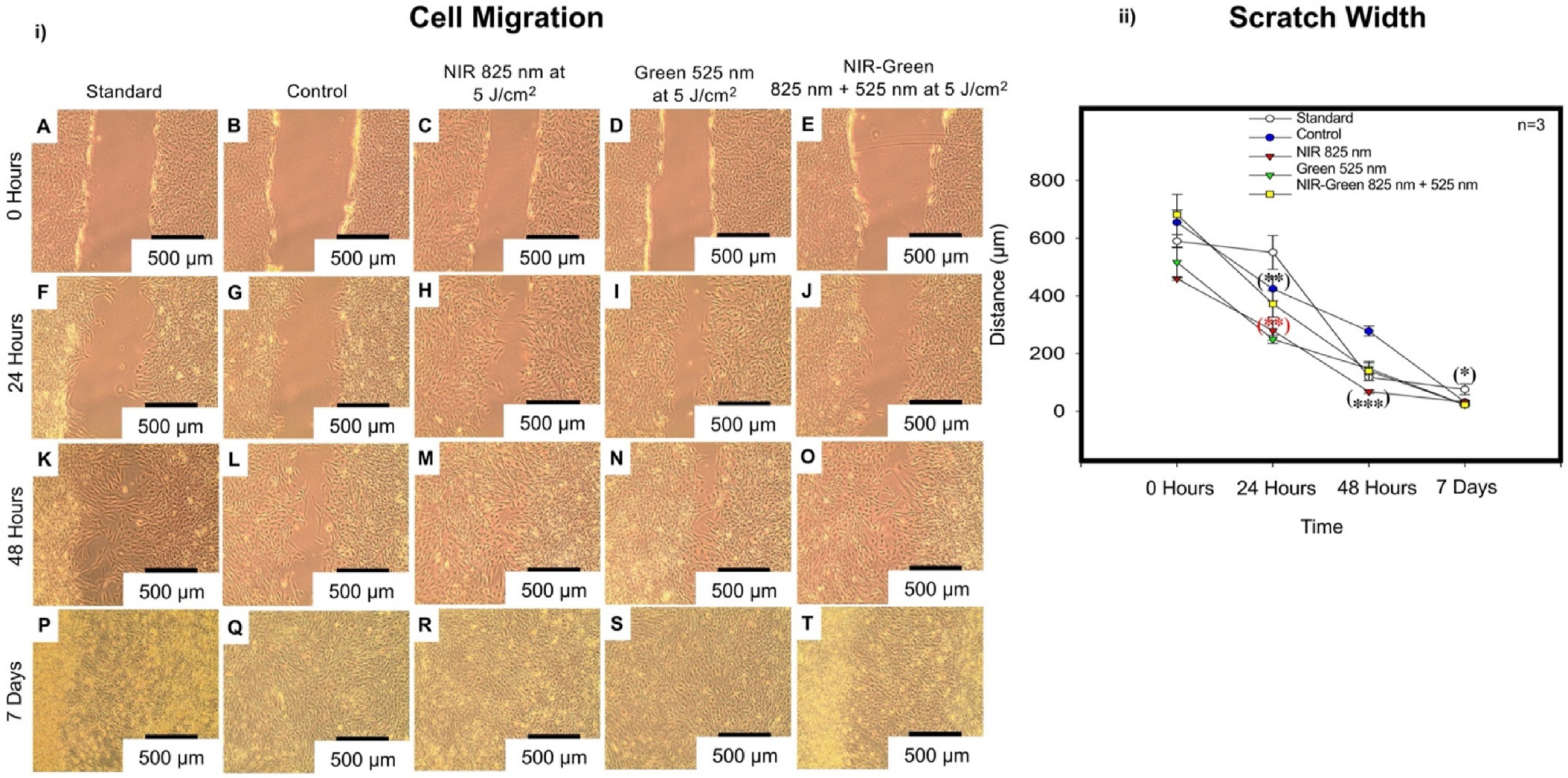
i) Morphological representation of cellular motility of differentiated immortalised adipose-derived mesenchymal stem cells. Cellular migration was visibly apparent by artificial wound closure at 24 h post-photobiomodulation treatment (H and I), at 48 h post-photobiomodulation treatment (K-M) and at 7 days post-photobiomodulation treatment (P-T). ii) Motility analysis of differentiated immortalised adipose-derived mesenchymal stem cells. A statistically significant enhancement of cell motility occurred at 24 h and 48 h post-photobiomodulation amongst the experimental photobiomodulation groups compared to the control. This is suggestive of an enhanced cell homing capacity. At 7 days post-photobiomodulation treatment, all the experimental photobiomodulation groups including the standard and the control presented with statistically significant increases in artificial wound closure. The wound closure identified in the standard group at 7 days post-photobiomodulation may be explained by cell overcrowding.

The ‘central scratch test’ method identified the cellular motility ability of osteogenic induction differentiation medium treated immortalised ADMSCs at 0 h, 24 h, 48 h, and 7 days post-PBM treatment (Fig. 10 i)). It is crucial to detect cell homing via cellular motility, to properly place transplanted ADMSCs during the transplantation process [63]. Differentiated immortalised ADMSCs demonstrated a significant cell migration (P < 0.01) at 24 h post-PBM amongst all PBM experimental groups compared to the standard and the control. All PBM experimental groups presented with a statistically significant (P < 0.001) enhancement of cell motility at 48 h post-PBM treatment. At 7 days post-PBM treatment, all cells including the standard presented with a statistically significant (P < 0.05) cell motility as visibly identified on the morphology with a wound closure. The statistically significant increases identified at 24 h and 48 h amongst all PBM experimental groups indicate an enhanced capacity of cell homing (Fig. 10 ii)).

#### 3.3.2 EdU cell proliferation and cycle analysis

**Fig. 11:**
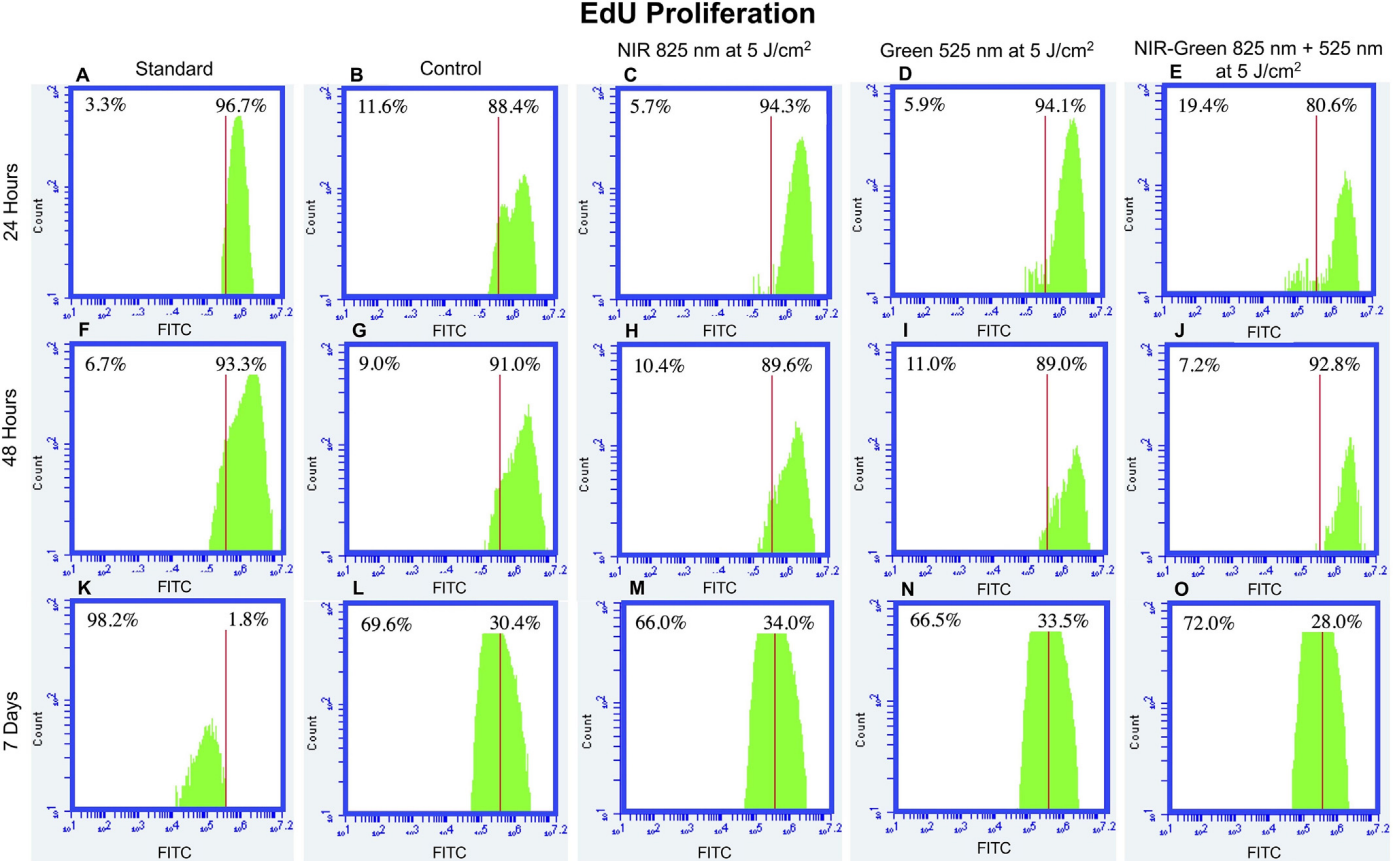
EdU Proliferation of osteogenic differentiated immortalised adipose-derived mesenchymal stem cells at 24 h, 48 h, and 7 days post-photobiomodulation treatment. A right shift occurred at 24 h and 48 h post-photobiomodulation treatment in EdU expression compared to the control group (C, D and J). A left shift occurred at 7 days post-photobiomodulation treatment in EdU expression compared to the control group (O). The left shift may be an outcome of cell culture contact competition due to cell over-crowding and a lack of available nutrients and/or the redirection of cellular energy for cellular differentiation.

EdU evaluates cell health and cycle analysis via DNA synthesis to determine cellular proliferation. Particularly, EdU measures the how much DNA is synthesized and replicated in the S phase. EdU served as an indicator of the proportion of cells in the S phase after being incorporated into the DNA of the samples during active DNA synthesis. The EdU results obtained identified a shift to the left or decreased EdU expression at 7 days post-PBM treatment amongst all cell groups including the standard (Fig. 11). This is suggestive that DNA synthesis has halted, and cells are no longer actively replicating, however, this was not necessarily induced by the osteogenic differentiation inducer presence nor PBM exposure. The decline in cellular proliferation may be explained by cell culture nutrient depletion [64] as the cells are over-crowded, thus, space and nutrients are limited in the cell culture plate by 7 days post-PBM treatment. Notably, a decrease in proliferation is also suggestive of cellular energy redirection for the use of cellular differentiation instead of for cellular proliferation [65].

#### 3.3.3 MTT cell proliferation/viability

**Fig. 12:**
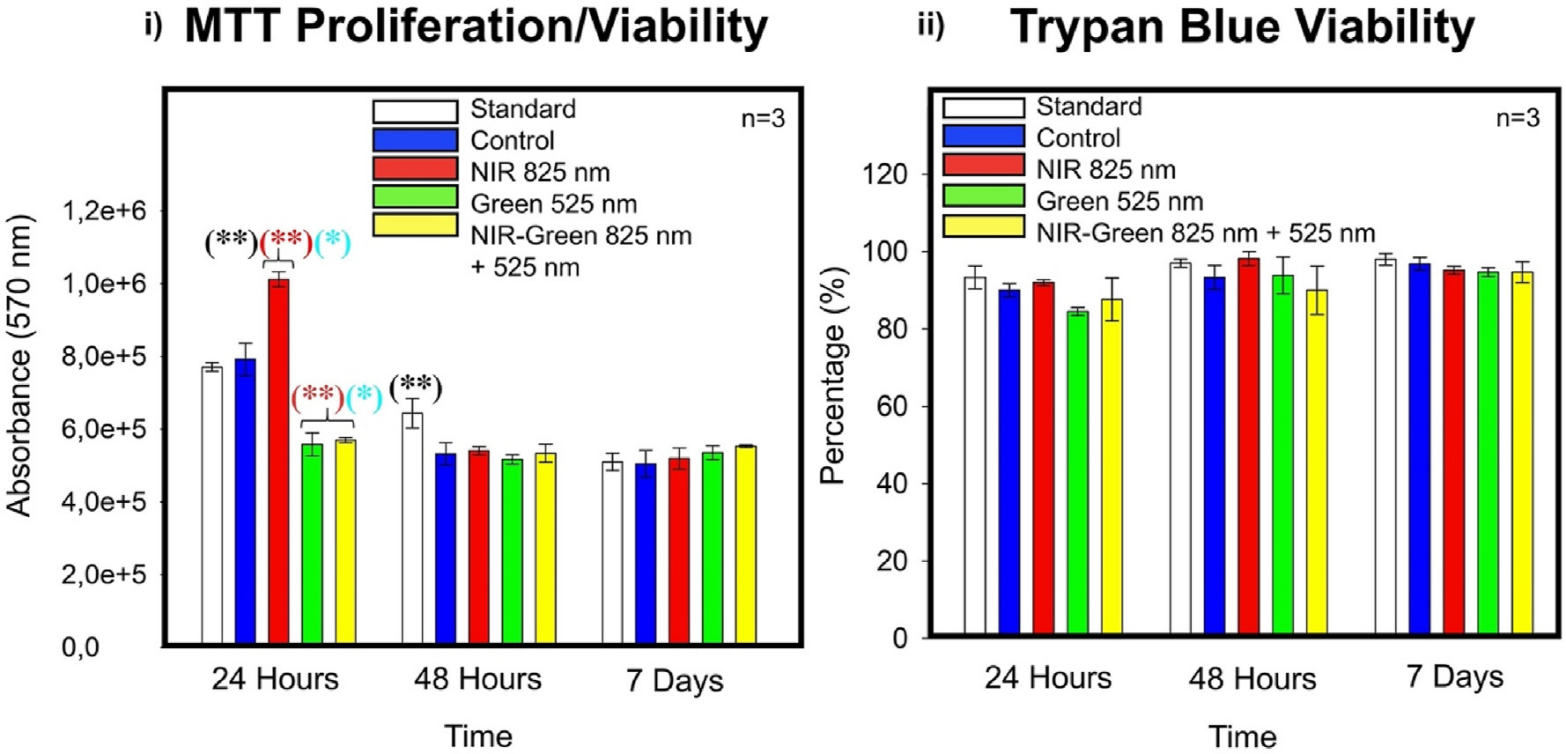
i) Cellular proliferation/viability analysis using the MTT assay demonstrated a statistically significant decline in cell proliferation at 24 h post-photobiomodulation treatment amongst the Green and Near Infrared-Green photobiomodulation groups compared to the control and Near-infrared photobiomodulation groups. The premature decline is suggestive of ATP redistribution from cellular proliferation to cellular differentiation. ii) Cellular viability using the trypan blue assay provided a reassurance of using photobiomodulation to differentiate immortalised adipose-derived mesenchymal stem cells whilst maintaining cell health as cell viability remained consistent at 24 h, 48 h, and 7 days post-photobiomodulation treatment.

The MTT assay did identify statistical significances in cell proliferation/viability amongst the standard (P < 0.01) at 24 h and 48 h and compared to the control (P < 0.01) at 24 h amongst all experimental PBM groups (Fig. 12 i)) was identified. Further statistical significances were identified amongst cells treated with NIR, G and NIR-G PBM wavelengths (P < 0.05) compared to one another (PBM groups) at 24 h (Fig. 12 i)). However, the noticeable decline is not suggestive of negative PBM repercussions on cell health as a decline was identified in the experimental control group at 48 h, which did not receive PBM treatment. The decline may be explained by an upsurge in cell proliferation resulting in contact competition amongst cells within the culture medium or the redirection of ATP for cellular proliferation to cellular differentiation. The overall cell health was assured with the trypan blue assay (Fig. 12 ii)) which demonstrated a consistent and maintained viability.

### 3.4 Cell cytotoxicity

#### 3.4.1 Mitochondrial membrane potential

**Fig. 13:**
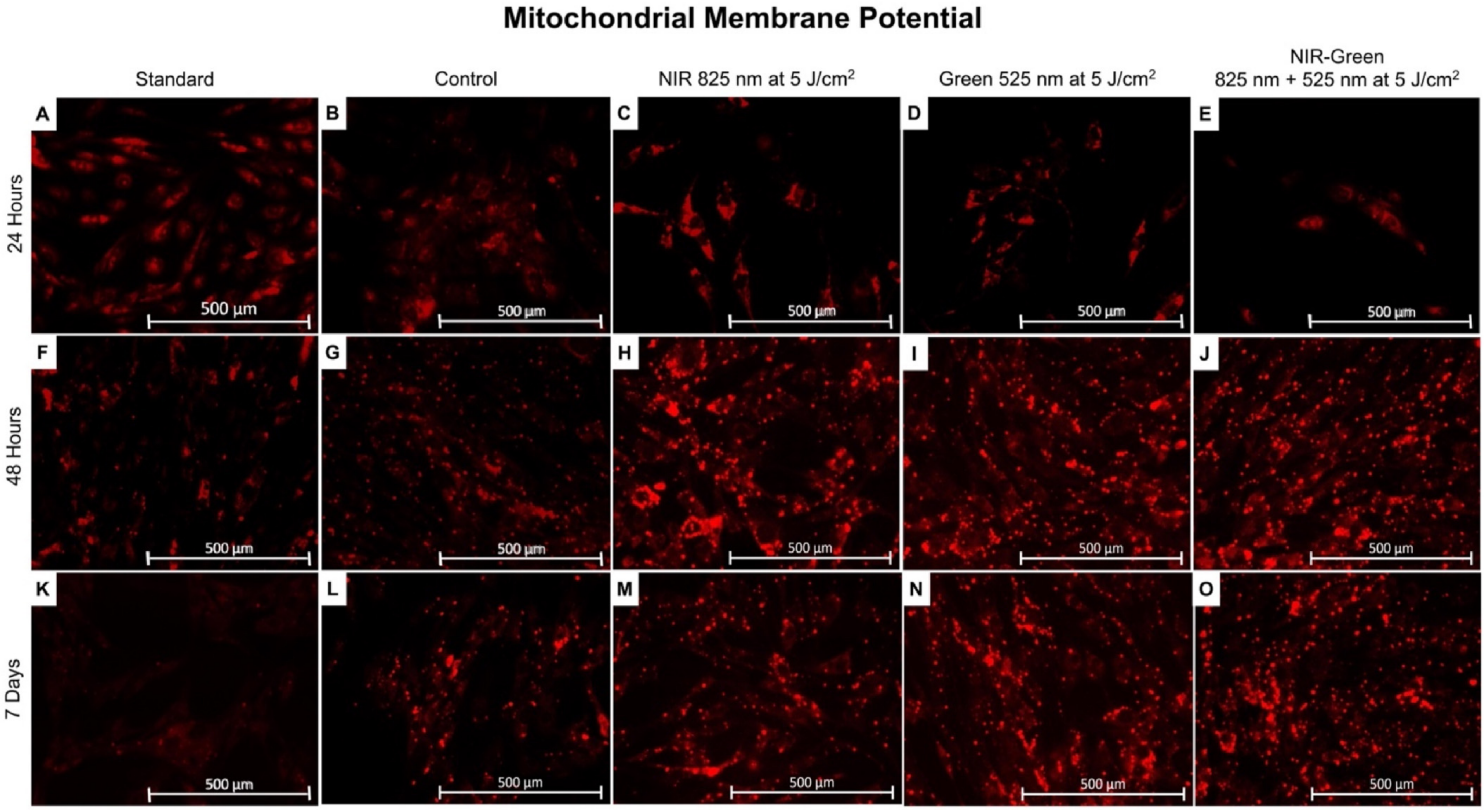
Morphological analysis of immortalised adipose-derived mesenchymal stem cell mitochondrial membrane potential at 24 h, 48 h, and 7 days post-photobiomodulation treatment. The cell morphology demonstrated an increase in fluorescence amongst the experimental photobiomodulation groups at 48 h post-photobiomodulation treatment (H-J). A further increase in fluorescence were identified in the control group and experimental photobiomodulation groups at 7 days post-photobiomodulation treatment (L-O), whereby, the Near-infrared-Green experimental group displayed fluorescence to a greater extent (O). The noticeable bright mitochondrial fluorescence is suggestive of cellular differentiation.

The MMP produced by proton pumps is a critical component of the energy storage pathway during oxidative phosphorylation. Mitochondrial membrane potential, in conjunction with the proton gradient, generates the transmembrane potential of hydrogen ions, which is used to synthesize cellular energy. Cells must maintain steady MMP levels to function normally, and any fluctuations observed are attributable to physiological activity [66]. This is true for PBM affecting the cells electron transport chain, where light absorption results in nitric oxide dissociating from CcO, stimulating the MMP [26]. Stem cell differentiation is guided by mitochondrial metabolism, where an increased MMP is associated with higher *in vitro* differentiation capacity [67], where studies have found that an upregulation of MMP is a prerequisite for cellular differentiation of MSCs [68] to satisfy an increased demand of cellular energy required for cellular differentiation [69]. The effect of osteogenic induction differentiation medium and NIR, G and NIR-G PBM wavelengths was investigated on MMP via immunofluorescent microscopy at 24 h, 48 h and 7 days post-PBM treatment (Fig. 13). Results show that cells maintained their MMP over time after irradiation with the cell mitochondria fluorescing a bright red. The fluorescent membrane probe that was used to stain the mitochondria showed an increase in MMP depicted by an increase in fluorescence seen specifically after 48 h and 7 days in the control groups and for all PBM treated samples. This is indicative that the induction type used may have caused lineage commitment. Furthermore, samples treated using PBM shows even brighter mitochondrial fluorescence, where the use of osteogenic induction differentiation medium and NIR-G PBM had the most significant increase suggesting immortalised ADMSC differentiation [69].

#### 3.4.2 Reactive oxygen species detection

**Fig. 14:**
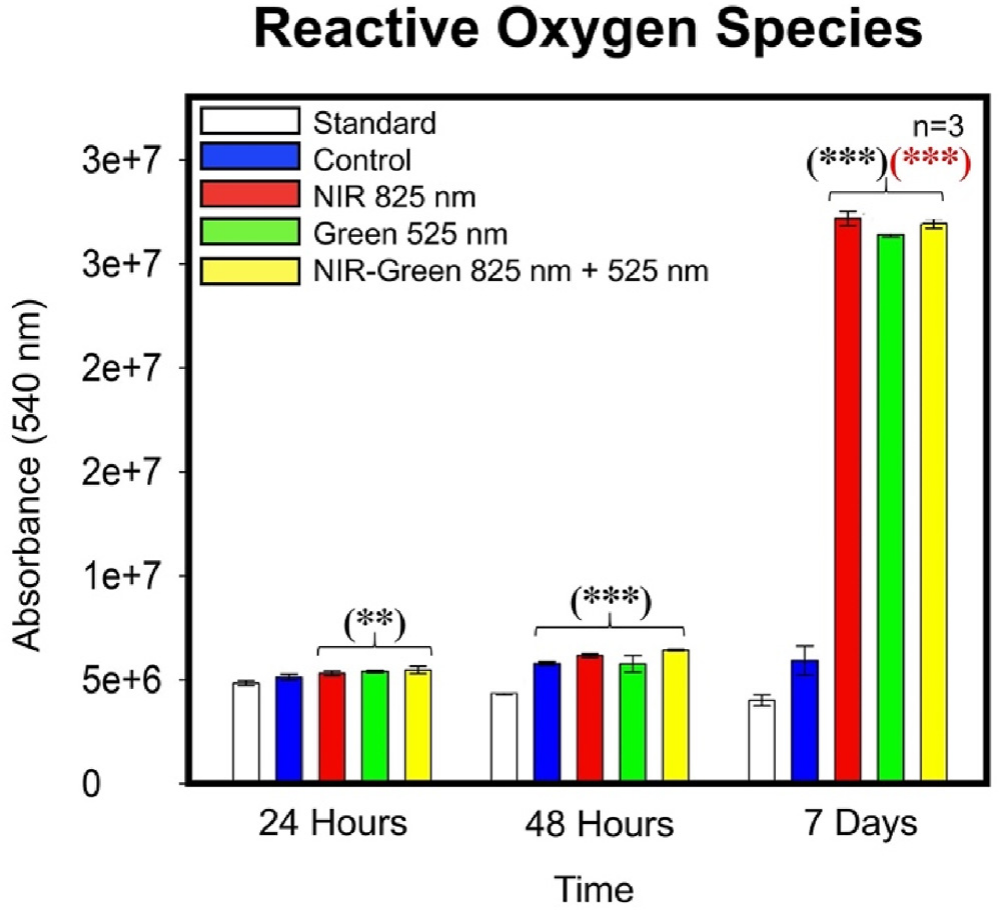
Reactive oxygen species production had displayed a significant increase amongst the experimental photobiomodulation groups at 24 h and 48 h post-photobiomodulation compared to the standard group and at 7 days post-photobiomodulation treatment in comparison to the control group. Due to the reassurance of consistent and sustainable cell viability despite photobiomodulation treatment (Fig. 12 ii), the significant increases in reactive oxygen species production are not suggestive of cell harm but instead, implies stem cell fate direction of differentiated immortalised adipose-derived mesenchymal stem cells.

A significant increase in ROS production was noted amongst cells treated with NIR, G and NIR-G PBM wavelengths compared to the standard cell group at 24 h, 48 h and 7 days post-PBM (Fig. 14). A statistically significant increase was identified in the NIR-G PBM group compared to the experimental control at 7 days post-PBM treatment. Notably, the increases in ROS production identified were not harmful to cells as the above investigative assays, such as MTT proliferation/viability assay and trypan blue viability assay as well as cellular morphology suggests the cells had remained healthy. Additionally, the cellular morphology further implied that the cellular health had not been impaired. Previous differentiation studies identified similar increases in ROS production and deemed these ROS concentrations to beneficially direct stem cell fate [70,71]. The slight increase in ROS is believed to be essential for cellular proliferation, differentiation, and survival outcomes [69].

## 4 Conclusion

Numerous studies have been conducted in an attempt to differentiate immortalised ADMSCs into osteoblast-like cells [3,36,56,72]. These studies discovered the results of NIR and G wavelengths independently, but none sought to combine these wavelengths to see if the result would produce both known investigational effects of proliferation and cellular differentiation. The findings identified all PBM groups in combination with osteogenic differentiation inducers, enhanced immortalised ADMSC differentiation into early osteoblast cell lines, with the NIR-G experimental group having enhanced the most successful differentiation outcome when compared to the NIR and G PBM groups. The following phenotypic and cellular changes were determined in the study as a result of the differentiation of immortalised ADMSCs into early osteoblasts using biochemical and molecular assays. The results of this study's characterisation and morphological analysis revealed that the combination of osteogenic differentiation inducers and PBM improves the differentiation of immortalised ADMSCs into early osteoblast-like cells. The increase of early osteogenic surface markers and of late osteogenic markers by PBM experimental groups suggest that immortalised ADMSCs have been redirected toward the early late-stage osteogenic cell lineage [56,72]. Morphological analysis with the Alizarin red stain revealed calcium deposit accumulation as early as 24 h after PBM treatment in the control and all PBM experimental groups, indicating the aid of differentiation inducers and PBM. However, the occurrence of calcium deposits was more visible in the PBM experimental groups. The biochemical analysis indicated a maintained cell viability, increased ROS levels indicative of cell fate redirection and an increased presence of MMP exposure. The identification of functional, differentiated osteoblast cells via ELISA protein expression analysis indicated an increased protein expression of early marker RUNX2 and late marker osteocalcin, specifically amongst the NIR-G PBM group supporting the suggestion that the combined wavelengths enhance cellular differentiation of immortalised ADMSCs into early osteoblast-like cells.

Therefore, the investigations of this study identified that using osteogenic differentiation inducers in conjunction with PBM improved the cellular differentiation of immortalised ADMSCs into early osteoblast cells. This study provided a scientifically sound basis for further *in vitro* investigations of immortalised ADMSC differentiation into osteoblast lineage cells using the newly combined effects of NIR-G PBM and osteogenic differentiation inducers. The analysis of this unique PBM wavelength combination with only a single fluency, as well as *in vitro* two-dimensional cell culture settings confining cell-to-cell interactions and cell adhesion and migration to one plane, are limitations of this study. Further research will be to determine the effects of this unique PBM combination, at varies fluences and within *in vitro* three-dimensional (3D) cell culture settings to better enhance immortalised ADMSC differentiation and proliferation. The beneficial inclusion of 3D cell culture techniques may possibly bridge a gap between *in vitro* and *in vivo* investigations for the speedy reach of clinical trials and the use of PBM in SC therapy.

## Author contributions

AC and HA - Conceptualization; DDS and AC - Methodology; DDS and AC - Validation; DDS - Formal analysis; DDS - Investigation; HA - Resources; DDS and AC - Writing - Original Draft; DDS, AC and HA - Writing - Review & Editing; DDS - Visualization; AC and HA - Supervision; DDS, AC and HA - Project administration; HA - Funding acquisition.

## Funding

This work was supported by the National Research Foundation of South Africa Thuthuka Instrument [Grant No. TTK2205035996]; the Department of Science and Innovation (DSI) funded African Laser Centre (ALC) [Grant No. HLHA23X task ALC-R007]; the University Research Council [Grant no. 2022URC00513]; the Department of Science and Technology's South African Research Chairs Initiative (DST-NRF/SARChI) [Grant No. 98337] and SARChI Doctoral Scholarship [Grant No. 146290].

## Data availability statement

The raw and analysed data used to support the findings of this study are available from the corresponding author upon request.

## Ethics statement

The study was reviewed and approved by the University of Johannesburg, Higher Degree Committee (HDC) and Research Ethics Committee (REC) clearance certificates (HDC-01-12-2020 and REC-471-2021).

## Declaration of competing interest

All authors declare that they have no conflicts of interest that could be perceived as influencing the research, data interpretation, or publication of this work. The funders have no involvement in the design, execution, or reporting of the research.

## References

[1] Johnell O., Kanis J.A. (2006). An estimate of the worldwide prevalence and disability associated with osteoporotic fractures. Osteoporos Int.

[2] Li C., Wei G., Gu Q., Wang Q., Tao S., Xu L. (2015). Proliferation and differentiation of rat osteoporosis mesenchymal stem cells (MSCs) after telomerase reverse transcriptase (TERT) transfection. Med Sci Mon Int Med J Exp Clin Res.

[3] Wang C., Meng H., Wang X., Zhao C., Peng J., Wang Y. (2016). Differentiation of bone marrow mesenchymal stem cells in osteoblasts and adipocytes and its role in treatment of osteoporosis. Med Sci Mon Int Med J Exp Clin Res.

[4] Coipeau P., Rosset P., Langonn A., Gaillard J., Delorme B., Rico A. (2009). Impaired differentiation potential of human trabecular bone mesenchymal stromal cells from elderly patients. Cytotherapy.

[5] Pai M.V. (2017). Osteoporosis prevention and management. J Obstet Gynaecol India.

[6] Yarom N., Yahalom R., Shoshani Y., Hamed W., Regev E., Elad S. (2007). Osteonecrosis of the jaw induced by orally administered bisphosphonates: incidence, clinical features, predisposing factors and treatment outcome. Osteoporos Int.

[7] Abrahamsen B., Eiken P., Eastell R. (2009). Subtrochanteric and diaphyseal femur fractures in patients treated with alendronate: a register-based national cohort study. J Bone Miner Res.

[8] Cosman F., de Beur S.J., LeBoff M.S., Lewiecki E.M., Tanner B., Randall S. (2014). Clinician's guide to prevention and treatment of osteoporosis. Osteoporos Int.

[9] Mason C., Dunnill P. (2008). A brief definition of regenerative medicine. Regen Med.

[10] Baer P.C., Geiger H. (2012). Adipose-derived mesenchymal stromal/stem cells: tissue localization, characterization, and heterogeneity. Stem Cell Int.

[11] Cawthorn W.P., Scheller E.L., MacDougald O.A. (2012). Adipose tissue stem cells: the great WAT hope. Trends Endocrinol Metabol.

[12] Dai R., Wang Z., Samanipour R., Koo K.I., Kim K. (2016). Adipose-derived stem cells for tissue engineering and regenerative medicine applications. Stem Cell Int.

[13] Fitzsimmons R.E.B., Mazurek M.S., Soos A., Simmons C.A. (2018). Mesenchymal stromal/stem cells in regenerative medicine and tissue engineering. Stem Cell Int.

[14] George S., Hamblin M.R., Abrahamse H. (2020). Photobiomodulation-induced differentiation of immortalized adipose stem cells to neuronal cells. Laser Surg Med.

[15] Kaur G., Dufour J.M. (2012).

[16] Paprocka M., Kraskiewicz H., Bielawska-Pohl A., Krawczenko A., Masłowski L., Czyżewska-Buczyńska A. (2021). From primary MSC culture of adipose tissue to immortalized cell line producing cytokines for potential use in regenerative medicine therapy or immunotherapy. Int J Mol Sci.

[17] Ito T., Sawada R., Fujiwara Y., Tsuchiya T. (2008). FGF-2 increases osteogenic and chondrogenic differentiation potentials of human mesenchymal stem cells by inactivation of TGF-β signaling. Cytotechnology.

[18] Kim H.K., Kim J.H., Abbas A.A., Kim D.O., Park S.J., Chung J.Y. (2009). Red light of 647 nm enhances osteogenic differentiation in mesenchymal stem cells. Laser Med Sci.

[19] Liu Z.J., Zhuge Y., Velazquez O.C. (2009). Trafficking and differentiation of mesenchymal stem cells. J Cell Biochem.

[20] Trentz O.A., Arikketh D., Sentilnathan V., Hemmi S., Handschin A.E., de Rosario B. (2010). Surface proteins and osteoblast markers: characterization of human adipose tissue-derived osteogenic cells. Eur J Trauma Emerg Surg.

[21] Jeon O., Rhie J.W., Kwon I.K., Kim J.H., Kim B.S., Lee S.H. (2008). In vivo bone formation following transplantation of human adipose-derived stromal cells that are not differentiated osteogenically. Tissue Eng.

[22] An C., Cheng Y., Yuan Q., Li J. (2010). IGF-1 and BMP-2 induces differentiation of adipose-derived mesenchymal stem cells into chondrocytes-like cells. Ann Biomed Eng.

[23] Lin Y., Wang T., Wu L., Jing W., Chen X., Li Z. (2007). Ectopic and in situ bone formation of adipose tissue-derived stromal cells in biphasic calcium phosphate nanocomposite. J Biomed Mater Res.

[24] Lee S.Y., Lee J.H., Kim J.Y., Bae Y.C., Suh K.T., Jung J.S. (2014). BMP2 increases adipogenic differentiation in the presence of dexamethasone, which is inhibited by the treatment of TNF-α in human adipose tissue-derived stromal cells. Cell Physiol Biochem.

[25] Anders J.J., Arany P.R., Baxter G.D., Lanzafame R.J. (2019). Light-emitting diode therapy and low-level light therapy are photobiomodulation therapy. Photobiomodul Photomed Laser Surg.

[26] de Freitas L.F., Hamblin M.R. (2016). Proposed mechanisms of photobiomodulation or low-level light therapy. IEEE J Sel Top Quant Electron.

[27] Akyol U.K., Sipal S., Demirci E., Gungormus M. (2015). The influence of low-level laser therapy with alendronate irrigation on healing of bone defects in rats. Laser Med Sci.

[28] de Andrade A.L.M., Luna G.F., Brassolatti P., Leite M.N., Parisi J.R., de Oliveira Leal Â.M. (2019). Photobiomodulation effect on the proliferation of adipose tissue mesenchymal stem cells. Laser Med Sci.

[29] Luiz A., Pinheiro B., Elizabeth M., Gerbi M.M. (2006). Photoengineering of Bone Repair Processes.

[30] Saygun I., Karacay S., Serdar M., Ural A.U., Sencimen M., Kurtis B. (2008). Effects of laser irradiation on the release of basic fibroblast growth factor (bFGF), insulin like growth factor-1 (IGF-1), and receptor of IGF-1 (IGFBP3) from gingival fibroblasts. Laser Med Sci.

[31] Chung H., Dai T., Sharma S.K., Huang Y.Y., Carroll J.D., Hamblin M.R. (2012). The nuts and bolts of low-level laser (Light) therapy. Ann Biomed Eng.

[32] George S., Hamblin M.R., Abrahamse H. (2022). Neuronal differentiation potential of primary and immortalized adipose stem cells by photobiomodulation. J Photochem Photobiol, B.

[33] Wang Y., Huang Y.Y., Wang Y., Lyu P., Hamblin M.R. (2016). Photobiomodulation (blue and green light) encourages osteoblastic-differentiation of human adipose-derived stem cells: role of intracellular calcium and light-gated ion channels. Sci Rep.

[34] Serrage H, Heiskanen V, Palin W, Cooper PR, Milward MR, Hadis M, et al. Under the spotlight: mechanisms of photobiomodulation concentrating on blue and green light. HHS Public Access. [n.d]. 10.1039/c9pp00089e PMC6685747 31183484

[35] Buscone S., Mardaryev A.N., Raafs B., Bikker J.W., Sticht C., Gretz N. (2017). A new path in defining light parameters for hair growth: discovery and modulation of photoreceptors in human hair follicle. Laser Surg Med.

[36] Fekrazad R., Asefi S., Eslaminejad M.B., Taghiar L., Bordbar S., Hamblin M.R. (2018).

[37] Huang Y.Y., Chen A.C.H., Carroll J.D., Hamblin M.R. (2009). Biphasic dose response in low level lightherapy. Dose Response.

[38] Ren F., Wang K., Zhang T., Jiang J., Nice E.C., Huang C. (2015). New insights into redox regulation of stem cell self-renewal and differentiation. Biochim Biophys Acta Gen Subj.

[39] Atashi F., Modarressi A., Pepper M.S. (2015). The role of reactive oxygen species in mesenchymal stem cell adipogenic and osteogenic differentiation: a review. Stem Cell Dev.

[40] Hamblin M.R., Huang Y.Y., Sharma S.K., Carroll J. (2011). Biphasic dose response in low level light therapy - an update. Dose Response.

[41] Sommer A.P., Pinheiro A.L.B., Mester A.R., Franke R.-P., Whelan H.T. (2001).

[42] George S., Hamblin M.R., Abrahamse H. (2018). Effect of red light and near infrared laser on the generation of reactive oxygen species in primary dermal fibroblasts. J Photochem Photobiol, B.

[43] Hamblin M.R., Huang Y.Y., Heiskanen V. (2019). Non-mammalian hosts and photobiomodulation: do all life-forms respond to light?. Photochem Photobiol.

[44] Tani A., Chellini F., Giannelli M., Nosi D., Zecchi-Orlandini S., Sassoli C. (2018). Red (635 nm), near-infrared (808 nm) and violet-blue (405 nm) photobiomodulation potentiality on human osteoblasts and mesenchymal stromal cells: a morphological and molecular in vitro study. Int J Mol Sci.

[45] Bölükbaşı Ateş G., Ak Can A., Gülsoy M. (2017). Investigation of photobiomodulation potentiality by 635 and 809 nm lasers on human osteoblasts. Laser Med Sci.

[46] Yin K., Zhu R., Wang S., Zhao R.C. (2017). Low-Level laser effect on proliferation, migration, and antiapoptosis of mesenchymal stem cells. Stem Cell Dev.

[47] Barboza Ca ugusto G., Ginani F., oura Soares D.M., Henriques Ac ristina G., Freitas R. de A. (2014). Low-level laser irradiation induces in vitro proliferation of mesenchymal stem cells. Einstein (Sao Paulo).

[48] Mvula B., Abrahamse H. (2016). Differentiation potential of adipose-derived stem cells when cocultured with smooth muscle cells, and the role of low-intensity laser irradiation. Photomed Laser Surg.

[49] Liao X., Li S.H., Xie G.H., Xie S., Xiao L.L., Song J.X. (2018). Preconditioning with low-level laser irradiation enhances the therapeutic potential of human adipose-derived stem cells in a mouse model of photoaged skin. Photochem Photobiol.

[50] Fekrazad R., Asefi S., Allahdadi M., Kalhori K.A.M. (2016). Effect of photobiomodulation on mesenchymal stem cells. Photomed Laser Surg.

[51] Escudero J.S.B., Perez M.G.B., de Oliveira Rosso M.P., Buchaim D.V., Pomini K.T., Campos L.M.G. (2019). Photobiomodulation therapy (PBMT) in bone repair: a systematic review. Injury.

[52] Stancker T.G., Vieira S.S., Serra A.J., do Nascimento Lima R., dos Santos Feliciano R., Silva J.A. (2018). Can photobiomodulation associated with implantation of mesenchymal adipose-derived stem cells attenuate the expression of MMPs and decrease degradation of type II collagen in an experimental model of osteoarthritis?. Laser Med Sci.

[53] Rosso MP. de O., Buchaim D.V., Kawano N., Furlanette G., Pomini K.T., Buchaim R.L. (2018). Photobiomodulation therapy (PBMT) in peripheral nerve regeneration: a systematic review. Bioengineering.

[54] de Oliveira H.A., Antonio E.L., Arsa G., Santana E.T., Silva F.A., Arruda D. (2018). Photobiomodulation leads to reduced oxidative stress in rats submitted to high-intensity resistive exercise. Oxid Med Cell Longev.

[55] Mansano B.S.D.M., da Rocha V.P., Antonio E.L., Peron D.F., do Nascimento De Lima R., Tucci P.J.F. (2021). Enhancing the therapeutic potential of mesenchymal stem cells with light-emitting diode: implications and molecular mechanisms. Oxid Med Cell Longev.

[56] Bölükbaşı Ateş G., Ak A., Garipcan B., Gülsoy M. (2020). Photobiomodulation effects on osteogenic differentiation of adipose-derived stem cells. Cytotechnology.

[57] Amaroli A., Agas D., Laus F., Cuteri V., Hanna R., Sabbieti M.G. (2018). The effects of photobiomodulation of 808 nm diode laser therapy at higher fluence on the in vitro osteogenic differentiation of bone marrow stromal cells. Front Physiol.

[58] Preciado S., Muntión S., Rico A., Pérez-Romasanta L.A., Ramos T.L., Ortega R. (2018). Mesenchymal stromal cell irradiation interferes with the adipogenic/osteogenic differentiation balance and improves their hematopoietic-supporting ability. Biol Blood Marrow Transplant.

[59] Niimi H., Ohsugi Y., Katagiri S., Watanabe K., Hatasa M., Shimohira T. (2020). Effects of low-level Er:YAG laser irradiation on proliferation and calcification of primary osteoblast-like cells isolated from rat calvaria. Front Cell Dev Biol.

[60] Yamauchi N., Taguchi Y., Kato H., Umeda M. (2018). High-power, red-light-emitting diode irradiation enhances proliferation, osteogenic differentiation, and mineralization of human periodontal ligament stem cells via ERK signaling pathway. J Periodontol.

[61] Lu T., Pei W., Wang K., Zhang S., Chen F., Wu Y. (2018). In vitro culture and biological properties of broiler adipose-derived stem cells. Exp Ther Med.

[62] Hong D., Chen H.X., Yu H.Q., Liang Y., Wang C., Lian Q.Q. (2010). Morphological and proteomic analysis of early stage of osteoblast differentiation in osteoblastic progenitor cells. Exp Cell Res.

[63] Koh K.S., Oh T.S., Kim H., Chung I.W., Lee K.W., Lee H.B. (2012). Clinical application of human adipose tissue-derived mesenchymal stem cells in progressive hemifacial atrophy (Parry-Romberg disease) with microfat grafting techniques using 3-dimensional computed tomography and 3-dimensional camera. Ann Plast Surg.

[64] Pavel M., Renna M., Park S.J., Menzies F.M., Ricketts T., Füllgrabe J. (2018). Contact inhibition controls cell survival and proliferation via YAP/TAZ-autophagy axis. Nat Commun.

[65] Heiden M.G.V., Cantley L.C., Thompson C.B. (1979). Understanding the warburg effect: the metabolic requirements of cell proliferation. Science.

[66] Zorova L.D., Popkov V.A., Plotnikov E.Y., Silachev D.N., Pevzner I.B., Jankauskas S.S. (2018). Mitochondrial membrane potential. Anal Biochem.

[67] Crous A., Rensburg M.J., Abrahamse H. Photobiomodulation Effects of Single Near-Infrared (825 nm), Green (525 nm) and Combination of Wavelengths on Adipose-Derived Mesenchymal Stem Cell Physiology n.d..

[68] Zhang Y., Marsboom G., Toth P.T., Rehman J. (2013). Mitochondrial respiration regulates adipogenic differentiation of human mesenchymal stem cells. PLoS One.

[69] Li Q., Gao Z., Chen Y., Guan M.X. (2017). The role of mitochondria in osteogenic, adipogenic and chondrogenic differentiation of mesenchymal stem cells. Protein Cell.

[70] Hu L., Yin C., Zhao F., Ali A., Ma J., Qian A. (2018). Mesenchymal stem cells: cell fate decision to osteoblast or adipocyte and application in osteoporosis treatment. Int J Mol Sci.

[71] Luo L., Hu D.H., Yin J.Q., Xu R.X. (2018). Molecular mechanisms of transdifferentiation of adipose-derived stem cells into neural cells: current status and perspectives. Stem Cell Int.

[72] Wang Y., Huang Y.Y., Wang Y., Lyu P., Hamblin M.R. (2017). Red (660 nm) or near-infrared (810 nm) photobiomodulation stimulates, while blue (415 nm), green (540 nm) light inhibits proliferation in human adipose-derived stem cells. Sci Rep.

